# Gestational age at birth, chronic conditions and school outcomes: a population-based data linkage study of children born in England

**DOI:** 10.1093/ije/dyac105

**Published:** 2022-05-19

**Authors:** Nicolás Libuy, Ruth Gilbert, Louise Mc Grath-Lone, Ruth Blackburn, David Etoori, Katie Harron

**Affiliations:** Institute of Health Informatics, University College London, London, UK; Centre for Longitudinal Studies, University College London, Social Research Institute, London, UK; Great Ormond Street Institute of Child Health, University College London, London, UK; Institute of Health Informatics, University College London, London, UK; Institute of Health Informatics, University College London, London, UK; Great Ormond Street Institute of Child Health, University College London, London, UK; Great Ormond Street Institute of Child Health, University College London, London, UK

**Keywords:** Gestational age, academic performance, special educational needs, chronic conditions, England, whole-population cohort

## Abstract

**Introduction:**

We aimed to generate evidence about child development measured through school attainment and provision of special educational needs (SEN) across the spectrum of gestational age, including for children born early term and >41 weeks of gestation, with and without chronic health conditions.

**Methods:**

We used a national linked dataset of hospital and education records of children born in England between 1 September 2004 and 31 August 2005. We evaluated school attainment at Key Stage 1 (KS1; age 7) and Key Stage 2 (KS2; age 11) and any SEN by age 11. We stratified analyses by chronic health conditions up to age 2, and size-for-gestation, and calculated population attributable fractions (PAF).

**Results:**

Of 306 717 children, 5.8% were born <37 weeks gestation and 7.0% had a chronic condition. The percentage of children not achieving the expected level at KS1 increased from 7.6% at 41 weeks, to 50.0% at 24 weeks of gestation. A similar pattern was seen at KS2. SEN ranged from 29.0% at 41 weeks to 82.6% at 24 weeks. Children born early term (37–38 weeks of gestation) had poorer outcomes than those born at 40 weeks; 3.2% of children with SEN were attributable to having a chronic condition compared with 2.0% attributable to preterm birth.

**Conclusions:**

Children born with early identified chronic conditions contribute more to the burden of poor school outcomes than preterm birth. Evaluation is needed of how early health characteristics can be used to improve preparation for education, before and at entry to school.

Key Messages

- Children born even a few weeks too early are less likely to achieve expected levels of attainment at age 7 and 11 and are more likely to have Special Educational Needs provision than those born at 40 weeks of gestation.
- This association is not fully explained by maternal risk factors including deprivation, age and parity, or by size-for-gestation at birth.
- Chronic conditions in school-aged children contribute more to the burden of Special Educational Needs and low academic attainment than preterm birth.
- Additional support prior to school entry to improve school readiness could be targeted at high-risk groups based on early health indicators shown to influence later outcomes.

## Introduction

Globally, around 11% of births are preterm (<37 weeks of gestation), but children born preterm account for a disproportionate amount of health care use (due to increased risk of chronic conditions) and childhood mortality.^1–3^ Rising numbers of children being born preterm and surviving to adulthood has led to an increased need to understand and improve long-term outcomes, including how education services can address their additional needs.^3–7^ However, there is a lack of evidence on how services meet the needs of preterm children across childhood. In addition, less attention has been given to children born early term (37–38 weeks), who also have worse health and developmental outcomes than those born at 39–40 weeks and are far more numerous than preterm births (approximately 24% early term versus 8% preterm in England).^8–10^ In the UK, approximately half of preterm births are due to obstetric intervention.^11^ Clinicians therefore have to weigh the benefits of hastening birth with the potential harms of being born too early.

Longitudinal cohort studies demonstrate that earlier gestational age at birth is associated with lower cognitive, motor and academic performance scores and more behavioural problems including attention-deficit hyperactivity disorder.^12^ Previous studies from the UK have shown a dose-response relationship between week of gestation and special educational needs (SEN) at school age.^9^^,^^13^ Being born too small, with weight at birth below the 10th centile, and having a chronic condition in early life add further risks of adverse outcomes.^14^^,^^15^ For example, a population-based study in Sweden found that those born small (versus normal) for gestation had poorer grade averages at age 16, irrespective of gestational age at birth.^12^^,^^16^ UK policy allows deferred entry to school for children born in the summer months (those born between April and August, who start school at a much younger age than the oldest in the year group who are born in September, at the start of term), but there is a lack of evidence on whether this should also take into account other factors, such as chronic conditions, which may also be related to school readiness.^17^

National data that are routinely collected in the UK and elsewhere on characteristics at birth and early hospitalizations could be used to predict and plan local- and national-level interventions to meet the additional needs of children born too early, too small or with chronic conditions. Early interventions before and during school can promote learning, socialization and participation in education, which in turn could have long-term benefits for the child, family and society.^18–21^ We used a linked national dataset, containing hospital and school records for all children in England, to evaluate school attainment at ages 7 and 11 and SEN, across the spectrum of gestational age and according to size for gestation and the presence of chronic conditions identified by age 2. We aimed to generate evidence to inform policy on provision of early support for children at risk of poorer outcomes at school age.

## Methods

### Data source

We used ECHILD (Education and Child Health Insights from Linked Data), a linked dataset containing information for pupils attending state schools in England from the National Pupil Database and information on hospital admissions from birth from Hospital Episode Statistics (HES).^22–24^ Linkage has been described in detail elsewhere.^25^ We used previous linkage of birth and delivery records in HES to obtain information from the ‘maternity tail’, including birthweight, gestational age, mode of delivery and parity.^26^

### Study population

The study population comprised children attending state schools in England (i.e. who were recorded in National Pupil Database) who were born between 1 September 2004 and 31 August 2005 and whose birth record was captured in HES. We excluded from the main analysis children with missing data on birthweight (86 702/451 773; 19.2%), gestational age (29.3%), parity and mode of delivery (0.4%), maternal age (0.01%) and deprivation (0.7%) and those with incomplete data on Key Stage 1 (KS1), Key Stage 2 (KS2) or SEN (Supplementary Figure S1, available as Supplementary data at *IJE* online). We also excluded those with invalid birthweight (<400 g or >5000 g; 1.9%) or gestational age (<24 weeks or >43 weeks of gestation at birth; 0.2%).

### Exposure

Gestational age in completed weeks was obtained from hospital birth records and was based on: (i) estimated date of delivery calculated by ultrasound scan measurements according to the trimester of the scan; (ii) estimated date of delivery measured from the first day of the last menstrual period; or (iii) clinical assessment [in the absence of (i) or (ii)].

We stratified analyses according to the presence of chronic health conditions, as captured in hospital admissions data up to age 2. We chose this age as a key period for identifying those in need of additional early developmental support, for example in early day care settings.^27–29^ Chronic conditions were identified based on the presence of diagnosis codes captured in hospital admission records using previously published code lists, grouped to account for small numbers of certain diagnoses before the age of 2 (see Supplementary Table S2, available as Supplementary data at *IJE* online).^30^ Admission records in HES allow the entry of up to 20 fields of clinical diagnoses coded using the International Statistical Classification of Diseases and Related Health Problems 10th Revision (ICD-10).

### Outcomes

We evaluated primary school attainment as measured at Key Stage 1 (KS1; age 7) and Key Stage 2 (KS2; age 11) in nationally mandated, universal assessments. At each Key Stage, we evaluated the percentage of children achieving the expected level of the National Curriculum for mathematics (Level 2 or above at KS1, Level 4 or above at KS2). We focused on mathematics, based on previous studies identifying more pronounced associations for mathematics than reading.^31^^,^^32^ We also evaluated the percentage of children who ever had SEN provision in primary school (defined as those with a statement of SEN or an Education Health & Care Plan or Action, Action Plus or Support) between the academic year 2010/11 (when our cohort were in reception class age 5) and 2015/16 (Year 6, age 11).^33^

### Risk factors

Delivery risk factors were coded according to HES maternity fields (Supplementary Table S1, available as Supplementary data at *IJE* online). Small or large for gestation (<10th or >90th percentile of birthweight for gestation) was derived from national birthweight percentiles.^34^ Mode of delivery was categorized as vaginal, caesarean or instrumental and was derived from the Office of Population Censuses and Surveys Classification of Interventions and Procedures codes, or delivery method as recorded in the maternity tail where no procedure code was available. Maternal age at delivery was categorized as <20, 20–24, 25–29, 30–34, 35–39, 40+ years. We also considered parity, sex, ethnic group (White, Black, Asian, Mixed or Other) and quintile of area deprivation at birth (Index of Multiple Deprivation).

### Statistical analysis

Relative risks for the association between week of gestation and SEN and school attainment at KS1 and KS2 were estimated using Poisson regression with robust standard errors.^35^ In multivariable models, we adjusted for all risk factors described above. In order to account for chronological age and to separate the effects of gestational age at birth and month of birth, we also adjusted for expected month of birth (based on estimated delivery date derived from subtracting gestational age at birth from 40 weeks, i.e. full term).^19^ In analyses of KS2 results, we adjusted for KS1 attainment, in order to determine whether the effects of gestational age persist through childhood.

To quantify the percentage of outcomes attributable to preterm births, early term births and the presence of chronic conditions, we estimated population attributable fractions (PAFs). The PAF represents the proportion of low attainment (or SEN) in the whole population, that can be attributed to the exposure (i.e. preterm birth, chronic condition) if a causal relationship can be assumed. All analyses were conducted using Stata V16.

### Sensitivity analyses

Due to missing data on birthweight (86 702/451 773; 19.2%), gestational age (29.3%), parity and mode of delivery (0.4%), maternal age (0.01%) and deprivation (0.7%), we performed a sensitivity analysis using multiple imputation by chained equations. The imputation models included all outcome variables, plus birthweight, gestational age, maternal age at delivery, parity, mode of delivery, sex, region and ethnic group. We combined results over 10 imputed datasets.

To address the fact that early birth is often related to obstetric intervention, we performed a sensitivity analysis restricting the cohort to spontaneous vaginal births. We also performed a sensitivity analysis where KS2 results were not adjusted for KS1 attainment, in order to see the overall effect of gestational age on KS2 attainment.

## Results

### Descriptive characteristics

Of 568 035 pupils born between 1 September 2004 and 31 August 2005 captured in the National Pupil Database data, 451 773 (80%) pupils were linked to their birth record in HES (Supplementary Figure S1). Of these, 306 717 had complete data on gestational age at birth and SEN.

Children born at lower gestations were more likely to be born small for gestation, to younger mothers and to live in more deprived areas (Table 1). Overall, 7.0% of the cohort had at least one chronic condition captured in hospital records before the age of 2; 0.9% had more than one chronic condition. The prevalence of chronic conditions increased with lower gestational age at birth: 6.1% of children born at 40 weeks had any chronic condition, compared with 38.8% for those born before 32 weeks (Table 1).

**Table 1:** Characteristics of study population by completed weeks of gestation at birth (*N *= 306 717)

	Very preterm		Moderately preterm		Late preterm		Early term		Term							
	24-32		32-33		34-36		37-38		39		40		41-43		All	
	n	(%)	n	(%)	n	(%)	n	(%)	n	(%)	n	(%)	n	(%)	n	(%)
**Total (row %)**	2141	0.7	2226	0.7	13 386	4.4	57 956	18.9	67 850	22.1	88 441	28.8	74 717	24.4	306 717	100.0
**Sex**																
Male	1165	54.4	1226	55.1	7303	54.6	30 233	52.2	34 500	50.8	44 477	50.3	37 761	50.5	156 665	51.1
Female	976	45.6	1000	44.9	6083	45.4	27 723	47.8	33 350	49.2	43 964	49.7	36 956	49.5	150 052	48.9
**Ethnic group**																
White	1571	73.4	1723	77.4	10 255	76.6	43 152	74.5	51 093	75.3	69 556	78.6	61 179	81.9	238 529	77.8
Asian	209	9.8	194	8.7	1454	10.9	7590	13.1	8192	12.1	8967	10.1	5662	7.6	32 268	10.5
Black	213	9.9	154	6.9	782	5.8	3326	5.7	3790	5.6	4383	5.0	3552	4.8	16 200	5.3
Any other ethnic group	14	0.7	24	1.1	172	1.3	793	1.4	1045	1.5	1232	1.4	842	1.1	4122	1.3
Mixed	134	6.3	131	5.9	723	5.4	3095	5.3	3730	5.5	4303	4.9	3482	4.7	15 598	5.1
**Parity**																
0	910	42.5	1016	45.6	5466	40.8	19 961	34.4	24 621	36.3	36 003	40.7	34 076	45.6	122 053	39.8
1	584	27.3	573	25.7	3750	28.0	18 725	32.3	23 065	34.0	29 189	33.0	22 799	30.5	98 685	32.2
2 or more	647	30.2	637	28.6	4170	31.2	19 270	33.2	20 164	29.7	23 249	26.3	17 842	23.9	85 979	28.0
**Size for gestation**																
Small (<10 centile)	243	11.3	211	9.5	1095	8.2	4449	7.7	5539	8.2	8355	9.4	7647	10.2	27 539	9.0
Normal	1712	80.0	1732	77.8	10 572	79.0	46 185	79.7	55 346	81.6	71 980	81.4	60 944	81.6	248 471	81.0
Large (>90 centile)	186	8.7	283	12.7	1719	12.8	7322	12.6	6965	10.3	8106	9.2	6126	8.2	30 707	10.0
**Mode of delivery**																
Spontaneous	877	41.0	1010	45.4	7963	59.5	34 869	60.2	45 356	66.8	65 099	73.6	49 182	65.8	204 356	66.6
Emergency c-section	1072	50.1	946	42.5	3297	24.6	7301	12.6	6234	9.2	9956	11.3	12 560	16.8	41 366	13.5
Elective c-section	143	6.7	163	7.3	1102	8.2	11 270	19.4	9830	14.5	2362	2.7	1798	2.4	26 668	8.7
Instrumental	49	2.3	107	4.8	1024	7.6	4516	7.8	6430	9.5	11 024	12.5	11 177	15.0	34 327	11.2
**Maternal age**																
<20	203	9.5	198	8.9	1134	8.5	3734	6.4	4587	6.8	6844	7.7	5951	8.0	22 651	7.4
20-24	446	20.8	459	20.6	2734	20.4	10 872	18.8	13 308	19.6	18 405	20.8	15 102	20.2	61 326	20.0
25-30	543	25.4	575	25.8	3297	24.6	14 418	24.9	17 549	25.9	23 014	26.0	19 579	26.2	78 975	25.7
30-34	518	24.2	592	26.6	3737	27.9	16 622	28.7	19 562	28.8	25 085	28.4	21 404	28.6	87 520	28.5
35-39	347	16.2	324	14.6	2025	15.1	9972	17.2	10 653	15.7	12 702	14.4	10 707	14.3	46 730	15.2
40-50	84	3.9	78	3.5	459	3.4	2338	4.0	2191	3.2	2391	2.7	1974	2.6	9515	3.1
**Quintile of deprivation at birth**																
Most deprived	756	35.3	778	35.0	4530	33.8	17 967	31.0	20 041	29.5	25 299	28.6	20 266	27.1	89 637	29.2
2	508	23.7	504	22.6	2920	21.8	12 445	21.5	14 505	21.4	18 995	21.5	16 366	21.9	66 243	21.6
3	356	16.6	355	15.9	2328	17.4	10 457	18.0	12 137	17.9	16 288	18.4	14 015	18.8	55 936	18.2
4	274	12.8	308	13.8	1882	14.1	8851	15.3	10 633	15.7	14 327	16.2	12 477	16.7	48 752	15.9
Most affluent	247	11.5	281	12.6	1726	12.9	8236	14.2	10 534	15.5	13 532	15.3	11 593	15.5	46 149	15.0
**Chronic conditions before age 2**																
Respiratory	396	18.5	78	3.5	271	2.0	849	1.5	785	1.2	956	1.1	708	0.9	4043	1.3
Metabolic/endocrine/digestive/renal/genitourinary	234	10.9	156	7.0	710	5.3	2256	3.9	2274	3.4	2703	3.1	2186	2.9	10 519	3.4
Neurological	344	16.1	123	5.5	378	2.8	1125	1.9	1021	1.5	1220	1.4	993	1.3	5204	1.7
Cardiovascular	294	13.7	67	3.0	221	1.7	476	0.8	330	0.5	407	0.5	328	0.4	2123	0.7
Any^a^	830	38.8	358	16.1	1457	10.9	4571	7.9	4417	6.5	5410	6.1	4356	5.8	21 399	7.0
Two or more^a^	353	16.5	78	3.5	264	2.0	631	1.1	507	0.7	552	0.6	470	0.6	2855	0.9

a Including behavioural conditions, cancer/blood disorders, chronic infections and musculoskeletal/skin conditions (see Supplementary Table S2, available as Supplementary data at *IJE* online).

### Association between gestational age and school attainment

Of 300 493 pupils with KS1 results, 8.6% of children did not achieve the expected level, ranging from 7.6% for children born at 41 weeks to 50.0% at 24 weeks (Table 2). A similar pattern was seen for the 294 170 children with KS2 results, although the percentage of children not achieving expected levels was higher (21.1% overall). The percentage of children not achieving expected levels at KS1 increased after 41 weeks of gestation.

**Table 2:** School attainment (Mathematics) and provision of Special Educational Needs^a^ support by week of gestation at birth

	Not achieving expected level at Key Stage 1^b^ (*n* = 300 493)		Not achieving expected level at Key Stage 2^b^ (*n* = 294 170)		Special Educational Needs^a^ (*n *= 306 717)	
Week of gestation	*n*	%	*n*	%	*n*	%
24	22	50.0	24	58.5	38	82.6
25	24	30.4	43	56.6	59	73.8
26	43	30.9	69	51.1	96	66.7
27	55	29.4	76	42.0	112	59.3
28	83	28.4	128	44.4	174	57.6
29	74	21.5	133	39.7	181	51.4
30	82	18.0	161	35.9	233	50.1
31	105	19.2	183	34.1	282	50.1
32	136	15.5	258	30.0	408	45.9
33	201	15.3	395	30.8	586	43.8
34	281	12.5	592	26.8	911	39.8
35	395	11.2	890	25.6	1438	39.8
36	939	12.8	1895	26.3	2851	38.1
37	1670	10.5	3684	23.7	5698	35.0
38	3794	9.3	8849	22.1	13 836	33.2
39	5532	8.3	13 549	20.8	21 132	31.1
40	6713	7.7	16 872	19.9	26 086	29.5
41	4581	7.6	11 775	20.0	17 820	29.0
42	937	7.9	2331	20.1	3648	30.0
43	90	8.3	243	22.8	356	32.2
All	25 757	8.6	62 150	21.1	95 945	31.3

a Special Educational Needs or an Education Health & Care Plan or Action, Action Plus or Support between Reception and Year 6.

b Key stage 1 refers to the first 2 years of the Educational National Curriculum in England, i.e. Years 1 and 2 (ages 5 to 7). Key stage 2 refers to Years 3 to 6 (ages 7 to 11).

Of children with any chronic condition recorded by age 2, 16.0% and 29.6% did not achieve the expected level at KS1 and KS2 respectively, compared with 7.8% and 19.6% for children with no chronic conditions (Supplementary Table S3, available as Supplementary data at *IJE* online). Among all children not achieving the expected level at KS1, 13.3% had a chronic condition (Supplementary Table S4, available as Supplementary data at *IJE* online). According to the PAF, 6.8% of children not achieving expected levels at KS1 was attributable to chronic conditions (Supplementary Table S5, available as Supplementary data at *IJE* online). Among children not achieving the expected level at KS2, 10.2% had a chronic condition and the PAF was 3.4%.

**Figure 1:**
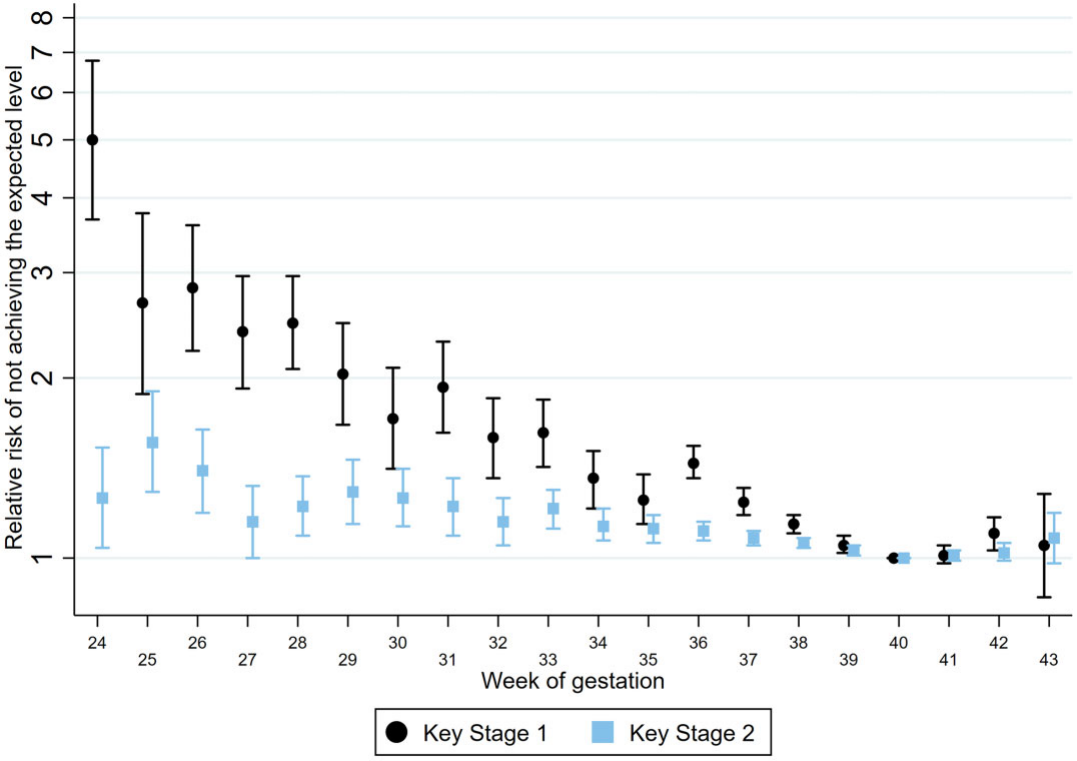
Association between gestational age at birth and school attainment at Key Stage 1 and Key Stage 2. Figure shows relative risk (log scale) comparing children born at each week of gestation with those at 40 weeks of gestation, adjusted for sex, parity, size of gestation, mode of delivery, maternal age, ethnic group, quintile of deprivation and expected month of delivery. KS2 results are adjusted for KS1 attainment: not achieving Level 2 at Key Stage 1 and Level 4 at Key Stage 2. Key Stage 1 refers to the first 2 years of the Educational National Curriculum in England, i.e. Years 1 and 2 (ages 5 to 7). Key Stage 2 refers to Years 3 to 6 (ages 7 to 11)

The association between gestational age and school attainment remained, but was attenuated, when adjusting for size-for-gestation and other risk factors (Figure 1; Supplementary Table S3). Children born to younger mothers, those living in deprived areas and those with older siblings were less likely to achieve expected levels (Supplementary Table S3). We also saw a clear pattern by month of birth, whereby summer-born children were less likely to achieve expected levels, likely due to their younger age at school start: children with an expected delivery month of August 2005 had an adjusted relative risk of 2.70 (95% CI 2.52, 2.89) for not achieving expected levels of attainment at Key Stage 1, compared with an expected delivery month of September 2004 (Supplementary Table S3). There was also a clear effect of birthweight: children who were born small-for-gestation were less likely to achieve the expected levels than those born normal- or large-for-gestation, across the range of gestational age (Supplementary Figure S3).

Early term births (37–38 weeks; 18.9% of the cohort) accounted for 21.2% of children not achieving expected levels at KS1 and 20.2% of children not achieving expected levels at KS2; children born preterm (5.8% of the cohort) accounted for a total of 9.5% at KS1 and 7.8% at KS2.

**Figure 2:**
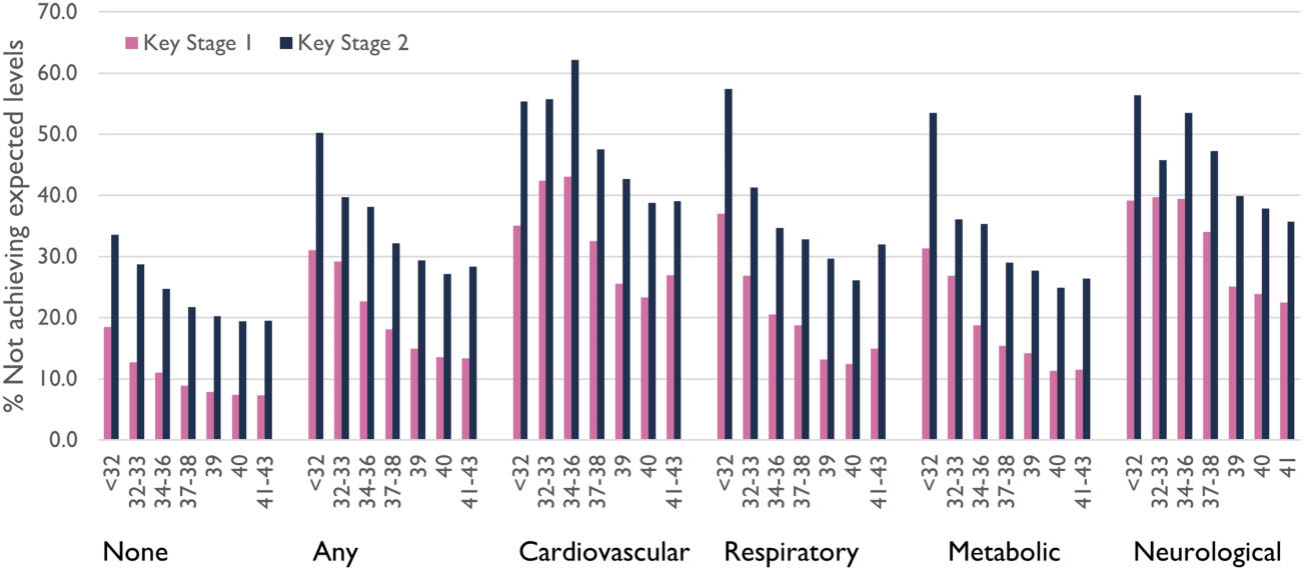
School attainment according to gestational age at birth and presence of chronic conditions by age 2. Key Stage 1 refers to the first 2 years of the Educational National Curriculum in England, i.e. Years 1 and 2 (ages 5 to 7). Key Stage 2 refers to Years 3 to 6 (ages 7 to 11)

The association between gestational age and attainment was consistent across chronic condition groups; children with neurological or cardiovascular conditions were least likely to achieve expected levels (Figure 2;Supplementary Figure S2, Supplementary Table S4, available as Supplementary data at *IJE* online).

### Association between gestational age and special educational needs

Overall, 31.3% of children had ever had SEN between reception Year 6, ranging from 82.6% at 24 weeks to 29.0% at 41 weeks (Table 2). Relative risks for SEN were 2.40 (95% CI 2.01, 2.87) for children born at 24 weeks, 1.35 (1.26, 1.45) for children born at 32 weeks and 1.13 (95% CI 1.10, 1.15) for children born at 37 weeks, compared with children born at 40 weeks (Supplementary Table S3). Children born to younger mothers, those living in more deprived areas, those with older siblings and those born later in the year were more likely to have SEN (Supplementary Table S3). Of children with any chronic condition recorded by age 2, 44.8% had SEN provision compared with 30.3% of children with no chronic conditions (Supplementary Table S3). Among all children receiving SEN support, 10.0% had a chronic condition (Supplementary Table S4). According to the PAF, 3.2% of the number of children with SEN was attributable to having a chronic condition (compared with 2.0% attributable to preterm birth, Supplementary Table S5).

**Figure 3:**
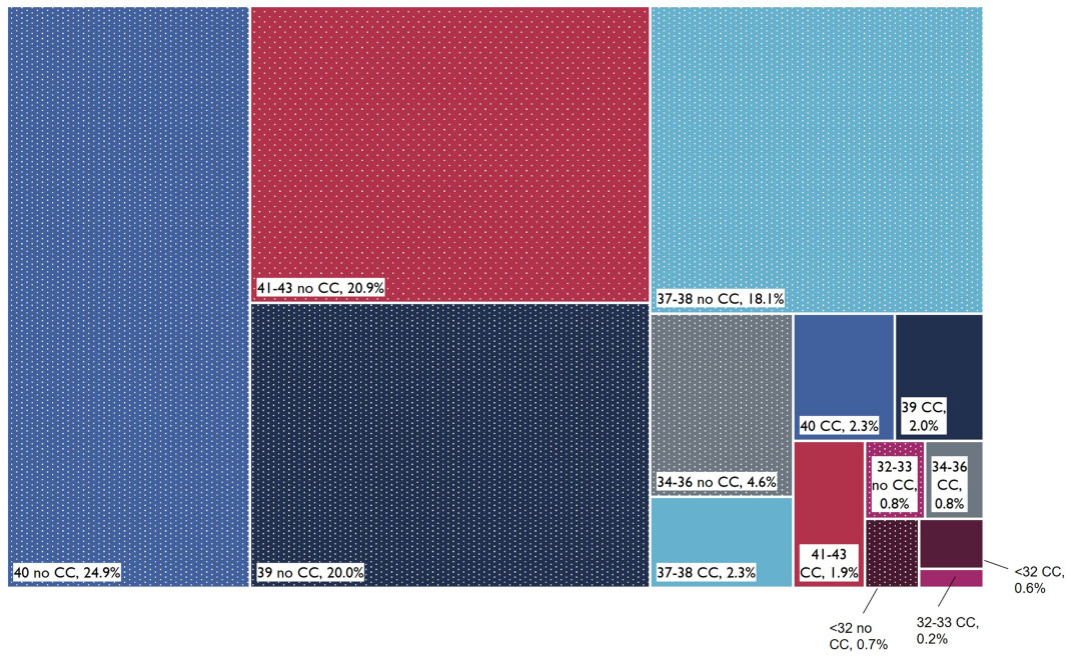
Breakdown of children with Special Educational Needs (SEN) according to week of gestation at birth and presence of chronic condition by age 2 (CC = any chronic condition; no CC = no chronic conditions). Together, the boxes reflect the total population of children with SEN (totalling 100%). Percentages show proportion of the population with SEN by gestational age in weeks (indicated by colour) and the presence (solid colour) or absence (textured colour) of chronic condition recorded by age 2. For example, 24.9% of children with SEN were born at 40 weeks of gestation and had no chronic condition

Early term births accounted for 20.4% of children with SEN whereas children born preterm accounted for a total of 7.7% (Figure 3).

The association between gestational age and SEN was consistent across chronic condition groups; children with neurological conditions were most likely to have SEN provision (Supplementary Table S4).

### Sensitivity analyses

We based the secondary (multiple imputation) analysis on the 451 773 children linked after exclusion restrictions. The results of the sensitivity analyses using multiple imputation (Supplementary Table S6, available as Supplementary data at *IJE* online), showed similar results to the main analysis, as did the analysis restricting to spontaneous vaginal births only (Supplementary Table S7, available as Supplementary data at *IJE* online). The results of our sensitivity analysis evaluating KS2 attainment without adjusting for prior KS1 attainment showed that the effects of gestational age persist beyond KS1 and are still apparent at age 11 (Supplementary Figure S4, available as Supplementary data at *IJE* online).

## Discussion

Our population-based cohort study fills an evidence gap by examining which school-age children are most at risk of not achieving expected levels of attainment, or of needing special educational needs support, on the basis of gestational age, size-for-gestation and presence of chronic conditions identified by age 2. We show, for the first time, that chronic conditions contribute more to the burden of SEN and low academic attainment than preterm birth (on the basis of population attributable fractions, 3.2% of children with SEN were attributable to having chronic conditions compared with 2.0% attributable to preterm birth). We add to previous evidence that children born early term (37–38 weeks) are more likely to have poor outcomes compared with those born at 40 weeks, and demonstrate that the dose-response relationship between gestational age at birth and school outcomes remains when accounting for size-for-gestation.^18–20^ Nevertheless, two-thirds of children receiving SEN provision were born at ≥39 weeks of gestation and had no chronic condition recorded in hospital records before age 2; 7.6% of the children did not achieve the expected level at KS1 (19.9% at KS2) and 29.0% had SEN provision despite ‘optimal’ gestation of 40–41 weeks.

A major strength of our study is that we used linked data from health and education that covered an entire cohort of children born in England in 1 year from September 2004. Using data from over 300 000 children gave us the statistical power to assess outcomes across each week of gestation, and to stratify by chronic condition. By using linkage of delivery and birth records, we were able to take account of maternal factors such as parity.^26^ Using routinely collected data on education and health outcomes means that our study is not affected by recall or ascertainment bias.

Limitations of our study include the small numbers of births in some categories, which meant that we were unable to look at more granular categories of chronic conditions. Like all observational studies, there may be residual confounding that explains the observed associations. Linking to information on maternal health, education or deprivation, for example, may further elucidate the mechanisms leading to both preterm birth and lower attainment.^36^ Missing outcome data might be related to exposures and may underestimate adverse outcomes for those born earliest or with the most severe conditions (since these children would be more likely to die, less likely to sit KS1 and KS2 tests and more likely to attend special schools or to not be assessed under the National Curriculum).^37^ Missing or invalid exposure data could lead to bias if missing data on birth characteristics depend on SEN/attainment after having taken into account other factors such as maternal age and deprivation. We addressed this through multiple imputation, and results were similar to the complete case analysis. A further limitation is that the children included in our study were born over 15 years ago, and so outcomes may not be generalizable to preterm infants being born today. We were also unable to analyse birth characteristics for children who did not have a birth record in HES, and so our analysis excludes children born outside England. However, our study provides a first exemplar of the research that will be possible using the ECHILD database, which links health and education data for all children born since 1995 in England.^24^

Although our study only included children in state schools in England (approximately 93% of pupils in this cohort), our results were consistent with findings of previous studies of cognitive and behavioural outcomes for preterm and post-term children, and are likely to be more widely generalizable.^9^^,^^13^^,^^16^^,^^38–45^ KS1 and KS2 are teacher assessments which may be prone to bias, particularly for SEN students.^46^ Educational attainment (and within that, mathematics as evaluated in this study) is only one aspect of a healthy and happy life and we do not currently capture other measures of ‘success’. However, quality of life is also reported to be lower in disability-free preterm compared with full-term children.^47^

Although rates of preterm birth are not declining, there has been progress in improving survival and health outcomes for children born preterm. Our study highlights three key challenges for improving school age outcomes for these children. First, we show that school outcomes are influenced by maternal characteristics such as age, parity and deprivation, which also predict adverse birth outcomes including preterm birth and low birthweight.^48^^,^^49^ Previous research has also shown that early social risk factors are as threatening as, and more common than, routinely documented biological risks.^50^ Support that facilitates healthy behaviours for the most vulnerable mothers, before pregnancy and early in pregnancy, could mitigate some of the common causes of these outcomes.^51^ Our findings also provide evidence to inform decisions about planned early births, particularly where this may lead to an increased risk of chronic conditions in the child. A balance of risks will need to be considered: approximately half of preterm deliveries are iatrogenic, meaning the delivery is due to obstetric intervention to avoid harm to the fetus, mother or both. Obstetricians need to balance these harms with developmental effects of being born too early.^52^

Second, we show that we can identify groups of children by age 2, based on gestational age and chronic conditions, who are likely to start school at a developmental disadvantage. This is particularly important for children who were born early term with chronic conditions, who are much more likely to have SEN than early-term children without chronic conditions (48.1% vs 32.5%, Supplementary Table S4). Additional support prior to school entry may also be particularly important for summer-born preterm children, who experience a ‘double disadvantage’ and may enter school more than a year behind some of their older peers, based on expected delivery date.^18–20^ Evidence shows that year of school entry modifies the impact of prematurity on school outcomes, and UK policy allows summer-born children to delay entry to school by a year.^17^ Schools and parents can also take into account which age group preterm children would have been born into (if they had been born full term) when deciding when a child should start school.^19^ Our findings provide additional evidence on the effects of having a chronic condition, which should also inform decisions about who should receive early help to enhance school readiness. Further work is needed to understand the impact of delayed school entry, or of being educated outside the normal year group, for preterm children born later in the year or for those with additional health needs.^19^

Third, our findings for outcomes at age 11 support previous evidence of cognitive deficits associated with lower gestational age persisting throughout childhood and beyond. Education is one of the major influences on outcomes across a child’s life course, and effective support during the first few years at school can help preterm children ‘catch up’.^12^^,^^20^ However, our findings highlight that preterm children are a heterogeneous group, and research is needed to understand how different interventions work for children with different chronic conditions (including for those born early term). Education professionals do not always have knowledge of the needs of children born preterm, and many feel ill-equipped to support them in school.^53^ Furthermore, standard early assessment tools such as the Ages & Stages Questionnaire at age 2–2½ are poorly discriminative of mild to moderate developmental deficits.^54^ Improved data sharing and linkage between health and education (as through the ECHILD Database) for pseudonymized, population-level research could therefore help early years services to understand which high-risk groups should be targeted, based on early health indicators and socioeconomic factors shown to influence later outcomes. Such data sharing could also inform development of interventions to improve educational outcomes for those with additional health needs, and to evaluate their effectiveness.^55^

## Ethics approval

Research ethics approval was granted (project ID 232547, REC reference 17/LO/1494) and data-sharing agreements are in place with NHS Digital (NIC-27404) and the Department for Education (DR150701.02). The Confidentiality Advisory Group confirmed that this research is exempt from review (reference 15/CAG/0004) because it only uses pseudonymized NHS data.

## Supplementary Material

dyac105_Supplementary_Data

## Data Availability

The data underlying this article cannot be shared publicly due to data-sharing agreements with NHS Digital and Department for Education.

## References

[1] Chawanpaiboon S , Vogel JP, Moller A-B et al Global, regional, and national estimates of levels of preterm birth in 2014: a systematic review and modelling analysis. Lancet Glob Health 2019;7:e37–46. 30389451 10.1016/S2214-109X(18)30451-0 PMC6293055

[2] Saigal S , Doyle LW. An overview of mortality and sequelae of preterm birth from infancy to adulthood. Lancet 2008;371:261–69. 18207020 10.1016/S0140-6736(08)60136-1

[3] Hack M , Taylor H, Drotar D et al Chronic conditions, functional limitations, and special health care needs of school-aged children born with extremely low-birth-weight in the 1990s. JAMA 2005;294:318–25. 16030276 10.1001/jama.294.3.318

[4] Teune MJ , Bakhuizen S, Gyamfi Bannerman C et al A systematic review of severe morbidity in infants born late preterm. Am J Obstet Gynecol 2011;205:374.e1–e9. 10.1016/j.ajog.2011.07.015 21864824

[5] Swamy GK , Ostbye T, Skjaerven R. Association of preterm birth with long-term survival, reproduction, and next-generation preterm birth. JAMA 2008;299:1429–36. 18364485 10.1001/jama.299.12.1429

[6] Costeloe KL , Hennessy EM, Haider S, Stacey F, Marlow N, Draper ES. Short term outcomes after extreme preterm birth in England: comparison of two birth cohorts in 1995 and 2006 (the EPICure studies). BMJ 2012;345:e7976. 23212881 10.1136/bmj.e7976 PMC3514472

[7] World Health Organization. *Born Too Soon: The Global Action Report on Preterm Birth.* Geneva, 2012. https://apps.who.int/iris/handle/10665/44864 (15 November 2021, date last accessed).

[8] NHS Digital. *NHS Maternity Statistics, 2019–20: HES NHS Maternity Statistics Tables* 2021. https://digital.nhs.uk/data-and-information/publications/statistical/nhs-maternity-statistics/2019-20 (29 November 2021, date last accessed).

[9] Alterman N , Johnson S, Carson C et al Gestational age at birth and child special educational needs: a UK representative birth cohort study. Arch Dis Child 2021;106:842–48 33483377 10.1136/archdischild-2020-320213 PMC7613205

[10] Harron K , Gilbert R, Cromwell D et al International comparison of emergency hospital use for infants: data linkage cohort study in Ontario and England. BMJ Qual Saf 2018;27:31–39. 10.1136/bmjqs-2016-006253 PMC5750429 28607037

[11] Smith GCS , Shah I, Pell JP et al Maternal obesity in early pregnancy and risk of spontaneous and elective preterm deliveries: a retrospective cohort study. Am J Public Health 2007;97:157–62. 17138924 10.2105/AJPH.2005.074294 PMC1716235

[12] Allotey J , Zamora J, Cheong-See F et al Cognitive, motor, behavioural and academic performances of children born preterm: a meta-analysis and systematic review involving 64 061 children. BJOG 2018;125:16–25. 29024294 10.1111/1471-0528.14832

[13] MacKay DF , Smith GCS, Dobbie R et al Gestational age at delivery and Special Educational Need: retrospective cohort study of 407,503 schoolchildren. PLoS Med 2010;7:e1000289. 20543995 10.1371/journal.pmed.1000289 PMC2882432

[14] Hu N , Fardell J, Wakefield CE et al School academic performance of children hospitalised with a chronic condition. Arch Dis Child 2022;107:289–96. 34475105 10.1136/archdischild-2020-321285 PMC8862027

[15] Wijlaars LPMM , Gilbert R, Hardelid P. Chronic conditions in children and young people: learning from administrative data. Arch Dis Child 2016;101:881–85. 27246068 10.1136/archdischild-2016-310716 PMC5050282

[16] Abel K , Heuvelman H, Wicks S et al Gestational age at birth and academic performance: population-based cohort study. Int J Epidemiol 2016;46:324–35. 10.1093/ije/dyw284 27818373

[17] Department for Education. *Admission of Summer-born Children: Advice for Local Authorities and School Admission Authorities.* 2021. https://www.gov.uk/government/publications/summer-born-children-school-admission/admission-of-summer-born-children-advice-for-local-authorities-and-school-admission-authorities (29 November 2021, date last accessed).

[18] Pettinger KJ , Kelly B, Sheldon TA et al Starting school: educational development as a function of age of entry and prematurity. Arch Dis Child 2020;105:160–65. 31409594 10.1136/archdischild-2019-317124 PMC7025727

[19] Odd D , Evans D, Emond A. Preterm birth, age at school entry and educational performance. PLoS One 2013;8:e76615. 24146899 10.1371/journal.pone.0076615 PMC3797787

[20] Odd D , Evans D, Emond A. Preterm birth, age at school entry and long term educational achievement. PLoS One 2016;11:e0155157. 27187690 10.1371/journal.pone.0155157 PMC4871348

[21] Lum A , Wakefield CE, Donnan B et al Facilitating engagement with school in students with chronic illness through positive education: a mixed-methods comparison study. Sch Psychol 2019;34:677–86. 31697153 10.1037/spq0000315

[22] Herbert A , Wijlaars LPMM, Zylbersztejn A et al Data Resource Profile: Hospital Episode Statistics Admitted Patient Care (HES APC). Int J Epidemiol 2017;46:1093. 28338941 10.1093/ije/dyx015 PMC5837677

[23] Jay MA , Mc Grath-Lone L, Gilbert R Data Resource Profile: The National Pupil Database. Int J Popul Data Sci 2019;4. 10.23889/ijpds.v4i1.1101 PMC7482519 32935030

[24] Mc Grath-Lone L , Libuy N, Harron K et al Data Resource Profile: The Education and Child Health Insights from Linked Data (ECHILD) database. Int J Epidemiol 2022;51:17. 34788413 10.1093/ije/dyab149 PMC8856003

[25] Libuy N , Gilbert R, Harron K et al Linking administrative education data to hospital data for four national cohorts of school pupils in England: methodology and evaluation of linkage quality. Int J Popul Data Sci 2021;6. 10.23889/ijpds.v6i1.1671 PMC8445153 34568585

[26] Harron K , Gilbert R, Cromwell DA et al Linking data for mothers and babies in de-identified electronic health data. PloS One 2016;11:e0164667. 27764135 10.1371/journal.pone.0164667 PMC5072610

[27] Kendall S , Nash A, Braun A et al *Evaluating the Use of a Population Measure of Child Development in the Health Child Programme: Two Year Review.* 2014. https://discovery.ucl.ac.uk/id/eprint/1493007/ (25 October 2021, date last accessed).

[28] Public Health England. *Best Start in Life and Beyond: Improving Public Health Outcomes for Children, Young People and Families.* Guidance to support the commissioning of the Healthy Child Programme 0–19: Health Visiting and school nursing services: Commissioning guide 2. 2018. https://assets.publishing.service.gov.uk/government/uploads/system/uploads/attachment_data/file/969168/Commissioning_guide_1.pdf (01 November 2021, date last accessed).

[29] Kendall S. *Evaluating the Use of a Population Measure of Children Development in the Healthy Child Programme Two Year Review.* 2014. https://kar.kent.ac.uk/54584/1/evaluating-the-use-of-a-population-measure-of-child-development-in-the-healthy-child-two-year-review.pdf (10 November 2021, date last accessed).

[30] Hardelid P , Dattani N, Gilbert R et al; on behalf of the Programme Board of the Royal College of Paediatrics and Child Health and the Child Death Overview Working Group. Estimating the prevalence of chronic conditions in children who die in England, Scotland and Wales: a data linkage cohort study. BMJ Open 2014;4:e005331. 10.1136/bmjopen-2014-005331 PMC4127921 25085264

[31] McBryde M , Fitzallen GC, Liley HG et al Academic outcomes of school-aged children born preterm: a systematic review and meta-analysis. JAMA Netw Open 2020;3:e202027. 32242904 10.1001/jamanetworkopen.2020.2027 PMC7125435

[32] Verfürden ML , Gilbert R, Lucas A et al Effect of nutritionally modified infant formula on academic performance: linkage of seven dormant randomised controlled trials to national education data. BMJ 2021;375:e065805. 34759005 10.1136/bmj-2021-065805 PMC8579423

[33] Jay MA , Gilbert R. Special educational needs, social care and health. Arch Dis Child 2021;106:83–85. 31969350 10.1136/archdischild-2019-317985

[34] Cole TJ , Statnikov Y, Santhakumaran S et al; on behalf of the Neonatal Data Analysis Unit and the Preterm Growth Investigator Group. Birth weight and longitudinal growth in infants born below 32 weeks’ gestation: a UK population study. Arch Dis Child Fetal Neonatal Ed 2014;99:F34–40. 23934365 10.1136/archdischild-2012-303536 PMC3888637

[35] Cummings P. Methods for estimating adjusted risk ratios. Stata J 2009;9:175–96.

[36] van Houdt CA , van Wassenaer-Leemhuis AG, Oosterlaan J et al Developmental outcomes of very preterm children with high parental education level. Early Hum Dev 2019;133:11–17. 31035105 10.1016/j.earlhumdev.2019.04.010

[37] Brownell M , Roos N, Fransoo R et al Is the class half empty? A population-based perspective on socioeconomic status and educational outcomes. Institute for Research on Public Policy Choices 2006;12:1–30.

[38] Lipkind HS , Slopen ME, Pfeiffer MR et al School-age outcomes of late preterm infants in New York City. Am J Obstet Gynecol 2012;206:222.e1–e6. 10.1016/j.ajog.2012.01.007 22381605

[39] Chyi LJ , Lee HC, Hintz SR et al School outcomes of late preterm infants: special needs and challenges for infants born at 32 to 36 weeks gestation. J Pediatr 2008;153:25–31. 18571530 10.1016/j.jpeds.2008.01.027

[40] Morse SB , Zheng H, Tang Y et al Early school-age outcomes of late preterm infants. Pediatrics 2009;123:e622–29. 19336353 10.1542/peds.2008-1405

[41] Larroque B , Ancel PY, Marret S et al Neurodevelopmental disabilities and special care of 5-year-old children born before 33 weeks of gestation (the EPIPAGE study): a longitudinal cohort study. Lancet 2008;371:813–20. 18328928 10.1016/S0140-6736(08)60380-3

[42] Larroque B , Ancel PY, Marchand-Martin L et al; Epipage Study group. Special care and school difficulties in 8-year-old very preterm children: the Epipage cohort study. PLoS One 2011;6:e21361. 21760892 10.1371/journal.pone.0021361 PMC3132214

[43] Eide MG , Øyen N, Skjærven R et al Associations of birth size, gestational age, and adult size with intellectual performance: evidence from a cohort of Norwegian men. Pediatr Res 2007;62:636–42. 17805203 10.1203/PDR.0b013e31815586e9

[44] Yang S , Platt RW, Kramer MS. Variation in child cognitive ability by week of gestation among healthy term births. Am J Epidemiol 2010;171:399–406. 20080810 10.1093/aje/kwp413 PMC3435092

[45] Johnson S , Hennessy E, Smith R et al Academic attainment and special educational needs in extremely preterm children at 11 years of age: the EPICure study. Arch Dis Child Fetal Neonatal Ed 2009;94:F283–89. 19282336 10.1136/adc.2008.152793

[46] Campbell T. Stereotyped at seven? Biases in teacher judgement of pupils’ ability and attainment. J Soc Pol 2015;44:517–47.

[47] Gire C , Resseguier N, Brévaut-Malaty V et al Quality of life of extremely preterm school-age children without major handicap: a cross-sectional observational study. Arch Dis Child 2019;104:333–39. 29960997 10.1136/archdischild-2018-315046

[48] Harron K , Verfuerden M, Ibiebele I et al Preterm birth, unplanned hospital contact, and mortality in infants born to teenage mothers in five countries: an administrative data cohort study. Paediatr Perinat Epidemiol 2020;34:645–54 . 32343005 10.1111/ppe.12685 PMC8425326

[49] Harron K , Gilbert R, Fagg J et al Associations between pre-pregnancy psychosocial risk factors and infants outcomes: a population-based cohort study in England. Lancet Public Health 2021;6:e97–105. 33516292 10.1016/S2468-2667(20)30210-3 PMC7848754

[50] Jutte DP , Brownell M, Roos NP et al Rethinking what is important: biologic versus social predictors of childhood health and educational outcomes. Epidemiology 2010;21:314–23. 20375842 10.1097/EDE.0b013e3181d61e61

[51] Barker M , Dombrowski SU, Colbourn T et al Intervention strategies to improve nutrition and health behaviours before conception. Lancet 2018;391:1853–64. 29673875 10.1016/S0140-6736(18)30313-1 PMC6075694

[52] Bentley JP , Roberts CL, Bowen JR et al Planned birth before 39 weeks and child development: a population-based study. Pediatrics 2016;138:e20162002. 10.1542/peds.2016-2002 27940704

[53] Johnson S , Gilmore C, Gallimore I et al The long-term consequences of preterm birth: what do teachers know? Dev Med Child Neurol 2015;57:571–77. 25586544 10.1111/dmcn.12683

[54] Sheldrick RC , Marakovitz S, Garfinkel D et al Comparative accuracy of developmental screening questionnaires. JAMA Pediatr 2020;174:366–74. 32065615 10.1001/jamapediatrics.2019.6000 PMC7042946

[55] Larose M-P , Haeck C, Ouellet-Morin I et al Childcare attendance and academic achievement at age 16 years. JAMA Pediatr 2021;175:939–46. 34096990 10.1001/jamapediatrics.2021.1192 PMC8185627

