# Supplementary material for: Gestational age at birth, chronic conditions and school outcomes: a population-based data linkage study of children born in England
Source: Int J Epidemiol. 2022 May 19;52(1):132–43. doi: 10.1093/ije/dyac105 (PMC9908051; doi:10.1093/ije/dyac105)
Supplement: dyac105_Supplementary_Data [file dyac105_supplementary_data.docx]

## Supplementary Material

**Coding of risk factors in HES**

**Parity**

The ‘numpreg’ field in HES records the number of previous pregnancies that resulted in registrable birth, live or stillborn. The ‘numpreg’ field takes the value between 0 to 19; 99 or blank fields are classified as missing. The longitudinal nature of HES allows us to inspect a mother’s delivery history and count her deliveries from the first to the current, provided that all the births happened in NHS hospitals. The method is not complete because any birth that happen outside the hospital are hard to trace. For instance, a birth outside the regional boundaries, such as in Scotland or in Wales. However, by using the existing information from HES this method does provide a way of distinguishing between primiparous women from multiparous women.

The following algorithm was applied to identify and correct inconsistent reporting of parity, or to provide a value when it was not known. Derived parity values include live and still births.

- Step 1: All of a mother’s deliveries records are sorted in date order.
- Step 2: Information from ICD-10 codes is used to determine whether a woman had a previous delivery:
  - O34.2 – maternal care due to uterine scar from previous surgery.
  - O75.7 – vaginal delivery following previous caesarean section.
  - Z35.4 – supervision of pregnancy with grand multiparity.
  - Z64.1 – problems related to multiparity.
  - Z87.5 – personal history of complications of pregnancy, childbirth and the puerperium
- Step 3: For any trust with more than 100 records of valid recorded parity, if the percentage of nulliparous women fell outside the range 20-70% then all parity data is set to missing.
- Step 4: Deriving parity:
  - For the first delivery the recorded parity in numpreg is assumed to be correct unless it is from a trust with data quality issues, in which case the value is reset to 99.
- If the first recorded parity in the HES field numpreg is codes as ‘0’, not known (‘99’) or blank (‘missing’) but the information from ICD-10 codes indicates a previous pregnancy (such as caesarean) then derived parity is set to ‘1’, otherwise it is set to ‘0’.
- For successive deliveries, parity is assumed to be one more than the last delivery unless the value of recorded numpreg field exceeds this, in which case the numpreg is assumed correct.
- Parity takes the value 0 = no previous pregnancies i.e. woman is first time mother (primiparous); 1 = one or more previous children (multiparous).

**Mode of Delivery**

Mode of delivery is recorded in the variable ‘delmeth’ in the HES maternity tail. OPCS-4 codes for any procedures undertaken are included in the core HES record and are available for most births. Since the mid-1990s, the procedure codes have been used for mode of birth in routine Maternity HES publications.

Mode of birth was therefore derived using the OPCS-4 procedure codes for records where the mode of birth was coded as missing in ‘delmeth’.

**Table S1.** Derivation of mode of birth using procedure codes in the HES delivery record, England

| **Code** | **OPCS-4 Code** | **Mode of birth** |
| --- | --- | --- |
| 0 | R24 | Spontaneous |
| 1 | R23 |  |
| 2 | R215 or R218 or R219 | Instrumental |
| 3 | R211 or R212 or R213 or R214 |  |
| 4 | R22 |  |
| 5 | R20 | Spontaneous |
| 6 | R19 | Instrumental |
| 7 | R17 | Elective Caesarean |
| 8 | R18 | Emergency Caesarean (and other surgical) |
| 9 | R25 | Missing |
| X | Otherwise |  |

**Table S2: ICD-10 Code lists used to identification diagnoses of chronic conditions in children up to age 2**

| **Chronic condition category** | **Types of conditions** | **Codes** |
| --- | --- | --- |
| Cardiovascular | Congenital heart disease, Other cardiovascular conditions | Q20-Q26, Q89.3 I00*-I28*, I31*-I39*, I41*, I42.0*-I42.5*, I42.7*-I42.9*, I43.0*, I43.1, I43.2*-I43.8*, I44.1*-I44.7*, I45.1*-I45.9*, I46*-I51*, I52.8, I70*-I71*, I72.1*-I72.4*, I72.8*, I72.9*, I73*-I77*, I79.0*, I79.1*, I79.8*, I81*-I82*, I98*-I99*, M03.6, N08.8, Q27, Q28, S26*, T82.0-T82.3, T82.5-T82.9, T86.2, Y60.5, Y61.5, Y62.5, Y84.0, Z45.0, Z50.0, Z94.1, Z95 |
| Respiratory | Asthma and chronic lower respiratory disease, Cystic fibrosis, Injuries, Congenital anomalies, Other respiratory conditions | J41-J47, E84, P75, S17*, S27*, S28*, T27*, T91.4*, Q30-Q37, Q79.0, G47.3, J60-J70, J80-J86, J96.1, J98, P27, Y55.6, Z43.0, Z93.0, Z94.2 |
| Metabolic/  endocrine/  digestive/renal  /genitourinary | Diabetes, Other endocrine conditions, Metabolic, Digestive, Renal/Genitourinary, Congenital anomalies of the digestive / renal / genitourinary system, Injuries, Other / unspecific | E10-E14, G59.0, G63.2, I79.2, M14.2, N08.3, O24, Y42.3, E00, E03.0, E03.1, E07.1, E22.0, E23.0, E25, E26.8, E29.1, E31, E34.1, E34.2, E34.5, E34.8, G13.2, G73.5, Y42.1, D55, E70-E72, E74-E78, E79.1-E79.9,E80.0-E80.3, E80.5, E80.7, E83, E85, E88.0, E88.1, E88.2*, E88.8, E88.9, G73.6, L99.0, M14.4, M14.3, N16.3, K20, K21.0, K22, K23.8, K25-K28, K29.0, K29.1, K29.3-K29.9, K31, K50-K52, K55, K57, K59.2, K63.0-K63.3, K66, K72-K76, K80-K83, K85.0, K85.1, K85.8, K85.9, K86.1-K86.9, K87.0, K90, M07.4, M07.5, M09.1, M09.2, T86.4, Z43.2-Z43.4, Z46.5, Z90.3, Z90.4, Z93.2-Z93.5, D63.8, G63.8, G99.8, I68.8, M90.8, N08.4, N00-N05, N07, N11-N15, N16.0, N16.2, N16.4, N16.5, N16.8, N18, N19, N20-N23, N25, N26, N28, N29, N31, N32, N33.8, N35, N36, N39.1, N39.3, N39.4, N40-N42, N70-N74, N80-N82, N85, N86*, N87,N88, P96.0, T82.4, T83.1, T83.2, T83.4-T83.9, T85.5, T86.1, Y60.2, Y61.2, Y62.2, Y84.1, Z49, Z93.6, Z94.0, Z99.2, Q38.0, Q38.3, Q38.4, Q38.6-Q38.8, Q39, Q40.2, Q40.3, Q40.8, Q40.9, Q41, Q42, Q43.1, Q43.3-Q43.7, Q43.9, Q44, Q45, Q50.0, Q51, Q52.0-Q52.2, Q52.4, Q54.0- Q54.3, Q54.8, Q54.9, Q55.0, Q55.5, Q56, Q60.1, Q60.2, Q60.4-Q60.6, Q61, Q62.0- Q62.6, Q62.8, Q63.0-Q63.2, Q63.8, Q63.9, Q64, Q79.2-Q79.5, Q87.8, Q89.1, Q89.2 Injuries S36*, S37*, S38*, S39.6*, S39.7*, T06.5*, T28*, T91.5*, E66, G63.3, G99.0, M14.5, N92*, Z86.3, Z93.8 |
| Neurological | Epilepsy, Cerebral palsy Injuries of brain, nerves, eyes or ears, Chronic eye conditions, Chronic ear conditions, Perinatal conditions, Congenital anomalies of neurological or sensory systems | F80.3, G40.0-G40.4, G40.6-G40.9, G41, R56.8, Y46.0-Y46.6, G80-G83, S05*-S08*, S12*, S14*, S24*, S34*, S44*, S54*, S64*, S74*, S84*, S94*, T06.0*- T06.2*, T26*, T90.4*, T90.5*, T91.1*, T91.3*, T92.4*, H05.1-H05.9, H13.3, H17, H18, H19.3, H19.8, H21, H26, H27, H28.0-H28.2, H31, H32.8, H33, H34, H35, H40, H42.0, H43, H44, H47, H54.0- H54.2, H54.4, T85.2, T85.3, Z44.2, H60.2, H65.2-H65.4, H66.1-H66.3, H69.0, H70.1, H73.1, H74.0-H74.3, H75.0, H80, H81.0, H81.4, H83.0, H83.2, H90.0, H90.3, H90.5, H90.6, H91, Z45.3, P10, P21.0, P52, P57, P90, P91.1, P91.2, P91.6, Q00-Q07, Q10.4, Q10.7, Q11-Q12, Q13.0-Q13.4, Q13.8, Q13.9, Q14-Q16, Q75.0, Q75.1, Q85, Q86.0, Q86.1, Q86.8, Q90-Q93, Q95.2, Q95.3, Q97, Q99 Other F02.2, F02.3,G00-G09, G10-G12, G13.8, G14, G20-G23, G24.1-G24.9, G25-G30, G31.0-G31.1, G31.8, G31.9, G32-G37, G43-G46, G47.0-G47.2, G47.4-G47.9, G50- G52, G53.0, G53.1, G53.8, G54, G55.8, G56-G58, G59.8, G60, G61, G62.0, G62.2- G62.9, G64, G70, G71,G72.2-G72.9, G73.0, G73.3, G90-G93, G94.2, G94.8, G95, G96, G98, G99.1, G99.2, I60-I67, I68.0, I68.2, I69, I72.0, I72.5, T85.0, T85.1, Y46.7- Y46.8, Z98.2 |
| Other | Behavioural / developmental disorders, Cancer / blood disorders, Chronic infections, Musculoskeletal | E24.4, F10-F19, F55*, G24.0*, G31.2, G40.5, G62.1, G72.0, G72.1, I42.6, K29.2, K70, K85.2, K85.3, K86.0, O35.4, R78.1*-R78.5*, Y47, Y49, Z50.2, Z50.3, Z71.4, Z71.5, Z72.2*, Z86.4, X60-X84, Y10-Y34†, Y87.0, Y87.2†, Z91.5, C00-C97, D00-D02, D05-D09, D12, D13, D14.1-D14.4, D15, D20, D32-D35, D37- D48, D63.0, E34.0, E88.3, G13.0, G13.1, G53.3, G55.0, G63.1, G73.1, G73.2, G94.1, M36.0, M36.1, M49.5, M82.0, M90.6, M90.7, N08.1, N16.1, Y43.1-Y43.3, Y84.2, Z08, Z51.0-Z51.2, Z54.1, Z54.2, Z85, Z86.0, Z92.3, F00-F01, F02.8, F03-F09, F20-F48, F50, F53, F54, F59*, F60-F69, F99*, Z09.3*, Z50.4*, Z86.5, Z91.4*, F70-F79, F80.0-F80.2, F80.8, F80.9, F81-F84, F88, F89, F90-F98, D80-D84, G53.2, Q98.0, D50*, D56.0-D56.2, D56.4, D56.8, D56.9, D57.0-D57.2, D57.8, D58, D61.0, D61.9, D64*, D66, D67, D68.0-D68.2, D68.4-D68.9, D69, D70-D76, M36.2-M36.4, M90.4, N08.2, Z86.2, B20-B24, F02.4, R75, Z21, A15-A19, E35.0, K23.0, K67.3, K93.0, M01.1, M49.0, P37.0 Other A50, A81, B18, B37.1, B37.5, B37.6, B37.7, B38.1, B39.1, B40.1, B44.0, B44.7, B45, B46, B48.7, B50.0, B50.8*, B51.0, B51.8*, B52.8*, B52.0, B55, B57.2-B57.5, B58.0, B59, B67, B69, B73, B74, B78.7, B90-B94, F02.1, K23.1, K93.1, M00, N33.0, P35.0- P35.2, P35.8, P35.9, P37.1, G55.1-G55.3, G63.5, G63.6, G73.7, J99.0, J99.1, L62.0, M05, M06, M07.0-M07.3, M07.6, M08, M09.8, M10-M13, M14.0, M14.6, M14.8, M30-M35, M40-M43, M45- M48,M50-M54, M60-M62, M63.8, M80.1-M80.9, M81.1-M81.9, M82.1, M82.8, M84.0-M84.2, M84.8, M84.9, M85, M86.3-M86.6, M89, M90.0, M91-M94, N08.5, Y45.4, S13*, S22.0*-S22.2*, S22.5*, S23*, S32*, S33*, S68.3*, S68.4*, S68.8*, S77*, S78*, S87*, S88*, S97*, S98.0*, S98.2*-S98.4*, T02*, T04*, T05*, T20.3*, T20.7*, T21.3*, T21.7*, T22.3*, T22.7*, T23.2*, T23.3*, T23.6*, T23.7*, T24.3*, T24.7*, T25.2*, T25.3*, T25.6*, T25.7*, T29.3*, T29.7*, T30.3*, T30.7*, T31.2*-T31.9*, T32.2*- T32.9*, T87.3-T87.6, T91.2* T91.8*, T92.6*, T93.1*, T93.4*, T93.6*, T94.0*, T94.1*, T95.0*, T95.1*, T95.4*, T95.8*, T95.9*, Y83.5, Z89.1, Z89.2, Z89.5-Z89.8, Z97.1, L10, L11.0, L11.8, L11.9, L12-L14, L28, L40-L45, L57, L58.1, L59, L87, L88, L90, L92, L95, L93, L98.5, M09.0, Q80, Q81, Q87.0-Q87.5, Q89.4, Q18.8, Q65.0-Q65.2, Q65.8, Q65.9, Q67.5, Q68.2, Q68.3*-Q68.5*, Q71-Q73, Q74, Q75.3-Q75.9, Q76.1-Q76.4, Q77, Q78, Q79.6, Q79.8, Q82.0-Q82.4, Q82.9, Q86.2, Q89.7-Q89.9 |

**Figure S1: Children included in study sample**

^ e.g. those who were not born in England

*This sample was used for the sensitivity analysis using multiple imputation

**Gender, birth weight, ethnic group, maternal age, quintile of deprivation, parity or mode of delivery.

***Descriptive summary provided in Table 1

**Table S3: School attainment at Key Stage 1 and attainment / SEN* at Key Stage 2, by gestational age at birth**

|  |  | Not achieving expected level (Level 2) at Key Stage 1 | | Not achieving expected level (Level 4) at Key Stage 2 | | SEN* | |
| --- | --- | --- | --- | --- | --- | --- | --- |
|  |  | Relative Risk (95% CI) | Adjusted** Relative Risk (95% CI) | Relative Risk (95% CI) | Adjusted** Relative Risk (95% CI) | Relative Risk (95% CI) | Adjusted** Relative Risk (95% CI) |
| Gestational age at birth (weeks) | 24 | 6.46 (4.80,8.69) | 5.00 (3.68,6.78) | 2.94 (2.27,3.81) | 1.26 (1.04,1.53) | 2.80 (2.45,3.20) | 2.40 (2.01,2.87) |
|  | 25 | 3.93 (2.81,5.48) | 2.67 (1.88,3.77) | 2.85 (2.34,3.47) | 1.56 (1.29,1.90) | 2.50 (2.19,2.85) | 2.00 (1.75,2.28) |
|  | 26 | 4.00 (3.11,5.13) | 2.83 (2.22,3.60) | 2.57 (2.18,3.03) | 1.40 (1.19,1.64) | 2.26 (2.01,2.54) | 1.92 (1.70,2.15) |
|  | 27 | 3.80 (3.04,4.75) | 2.39 (1.92,2.96) | 2.11 (1.78,2.51) | 1.15 (1.00,1.32) | 2.01 (1.78,2.26) | 1.53 (1.36,1.73) |
|  | 28 | 3.67 (3.06,4.41) | 2.47 (2.07,2.96) | 2.24 (1.96,2.55) | 1.22 (1.09,1.37) | 1.95 (1.77,2.15) | 1.56 (1.42,1.72) |
|  | 29 | 2.78 (2.27,3.41) | 2.03 (1.67,2.47) | 2.00 (1.75,2.28) | 1.29 (1.14,1.46) | 1.74 (1.57,1.93) | 1.43 (1.29,1.59) |
|  | 30 | 2.33 (1.91,2.84) | 1.71 (1.41,2.08) | 1.80 (1.59,2.04) | 1.26 (1.13,1.41) | 1.70 (1.55,1.86) | 1.41 (1.29,1.54) |
|  | 31 | 2.48 (2.08,2.95) | 1.93 (1.62,2.30) | 1.71 (1.52,1.93) | 1.22 (1.09,1.36) | 1.70 (1.56,1.85) | 1.47 (1.35,1.59) |
|  | 32 | 2.01 (1.72,2.34) | 1.59 (1.36,1.85) | 1.51 (1.36,1.67) | 1.15 (1.05,1.26) | 1.56 (1.45,1.67) | 1.35 (1.26,1.45) |
|  | 33 | 1.97 (1.73,2.24) | 1.62 (1.42,1.84) | 1.55 (1.42,1.68) | 1.21 (1.12,1.30) | 1.49 (1.40,1.58) | 1.32 (1.24,1.40) |
|  | 34 | 1.62 (1.45,1.81) | 1.36 (1.21,1.51) | 1.35 (1.26,1.45) | 1.13 (1.07,1.21) | 1.35 (1.28,1.42) | 1.21 (1.15,1.27) |
|  | 35 | 1.45 (1.32,1.59) | 1.25 (1.14,1.38) | 1.29 (1.21,1.36) | 1.12 (1.06,1.18) | 1.35 (1.30,1.41) | 1.23 (1.18,1.28) |
|  | 36 | 1.66 (1.55,1.76) | 1.44 (1.36,1.54) | 1.32 (1.27,1.38) | 1.11 (1.07,1.15) | 1.29 (1.25,1.33) | 1.19 (1.15,1.22) |
|  | 37 | 1.36 (1.29,1.43) | 1.24 (1.18,1.31) | 1.19 (1.15,1.23) | 1.08 (1.05,1.11) | 1.19 (1.16,1.22) | 1.13 (1.10,1.15) |
|  | 38 | 1.20 (1.15,1.25) | 1.14 (1.10,1.18) | 1.11 (1.09,1.14) | 1.06 (1.04,1.08) | 1.13 (1.11,1.14) | 1.09 (1.08,1.11) |
|  | 39 | 1.08 (1.04,1.11) | 1.05 (1.02,1.09) | 1.05 (1.03,1.07) | 1.03 (1.01,1.05) | 1.06 (1.04,1.07) | 1.05 (1.03,1.06) |
|  | 40 | **Ref** | **Ref** | **Ref** | **Ref** | **Ref** | **Ref** |
|  | 41 | 0.98 (0.95,1.02) | 1.01 (0.98,1.05) | 1.01 (0.98,1.03) | 1.01 (0.99,1.03) | 0.98 (0.97,1.00) | 1.00 (0.98,1.01) |
|  | 42 | 1.02 (0.95,1.09) | 1.10 (1.03,1.17) | 1.02 (0.97,1.05) | 1.02 (0.99,1.06) | 1.02 (0.99,1.05) | 1.05 (1.02,1.08) |
|  | 43 | 1.07 (0.88,1.31) | 1.05 (0.86,1.28) | 1.14 (1.02,1.28) | 1.08 (0.98,1.19) | 1.09 (1.00,1.19) | 1.07 (0.98,1.16) |
| Sex | Male |  | 1.40 (1.36,1.43) |  | 1.04 (1.03,1.05) |  | 1.7 (1.68,1.72) |
|  | Female |  | **Ref** |  | **Ref** |  | **Ref** |
| Previous births | 0 |  | **Ref** |  | **Ref** |  | **Ref** |
|  | 1 |  | 1.14 (1.11,1.17) |  | 1.09 (1.07,1.11) |  | 1.11 (1.09,1.12) |
|  | 2+ |  | 1.59 (1.54,1.63) |  | 1.26 (1.24,1.28) |  | 1.39 (1.37,1.41) |
| Size for gestation | Small |  | 1.55 (1.50,1.60) |  | 1.20 (1.17,1.22) |  | 1.26 (1.24,1.28) |
|  | Normal |  | **Ref** |  | **Ref** |  | **Ref** |
|  | Large |  | 0.81 (0.78,0.85) |  | 0.93 (0.91,0.95) |  | 0.93 (0.91,0.95) |
| Mode of delivery | Spontaneous vaginal |  | **Ref** |  | **Ref** |  | **Ref** |
|  | Emergency c-section |  | 1.05 (1.01,1.08) |  | 1.01 (0.99,1.03) |  | 1.01 (1.00,1.03) |
|  | Elective c-section |  | 1.04 (1.00,1.09) |  | 1.02 (1.00,1.05) |  | 1.00 (0.98,1.02) |
|  | Instrumental |  | 0.90 (0.87,0.94) |  | 0.94 (0.91,0.96) |  | 0.93 (0.91,0.95) |
| Maternal age (years) | <20 |  | 1.84 (1.76,1.92) |  | 1.17 (1.28,1.34) |  | 1.48 (1.45,1.51) |
|  | 20-24 |  | 1.36 (1.32,1.41) |  | 0.87 (1.15,1.19) |  | 1.24 (1.22,1.26) |
|  | 25-29 |  | **Ref** |  | **Ref** |  | **Ref** |
|  | 30-34 |  | 0.84 (0.81,0.87) |  | 0.87 (0.85,0.89) |  | 0.87 (0.85,0.88) |
|  | 35-39 |  | 0.87 (0.84,0.91) |  | 0.87 (0.85,0.89) |  | 0.87 (0.85,0.89) |
|  | 40-50 |  | 1.02 (0.95,1.09) |  | 0.93 (0.89,0.97) |  | 0.91 (0.88,0.94) |
| Ethnic group | White |  | **Ref** |  | **Ref** |  | **Ref** |
|  | Asian |  | 0.88 (0.85,0.91) |  | 0.71 (0.69,0.73) |  | 0.82 (0.81,0.84) |
|  | Black |  | 1.03 (0.98,1.08) |  | 0.85 (0.82,0.87) |  | 1.03 (1.01,1.05) |
|  | Other |  | 0.98 (0.89,1.08) |  | 0.74 (0.70,0.79) |  | 0.95 (0.91,0.99) |
|  | Mixed |  | 0.89 (0.85,0.94) |  | 0.91 (0.89,0.94) |  | 0.92 (0.90,0.94) |
| Quintile of Index of Multiple Deprivation | Most deprived |  | 1.43 (1.38,1.48) |  | 1.04 (1.02,1.06) |  | 1.24 (1.22,1.26) |
|  | 2 |  | 1.16 (1.12,1.20) |  | 1.01 (0.99,1.03) |  | 1.10 (1.09,1.12) |
|  | 3 |  | **Ref** |  | **Ref** |  | **Ref** |
|  | 4 |  | 0.84 (0.81,0.88) |  | 0.99 (0.97,1.01) |  | 0.90 (0.89,0.92) |
|  | Most affluent |  | 0.71 (0.67,0.75) |  | 0.93 (0.90,0.95) |  | 0.81 (0.80,0.83) |
| Not achieving expected level at Key Stage 1 |  |  |  |  | 5.01 (4.96,5.07) |  |  |
| Month of expected delivery date | Aug-04 |  | 0.84 (0.65,1.07) |  | 1.05 (0.94,1.17) |  | 1.00 (0.91,1.09) |
|  | Sep-04 |  | **Ref** |  | **Ref** |  | **Ref** |
|  | Oct-04 |  | 1.10 (1.01,1.18) |  | 1.02 (0.98,1.06) |  | 1.02 (0.99,1.05) |
|  | Nov-04 |  | 1.22 (1.13,1.32) |  | 1.05 (1.01,1.08) |  | 1.09 (1.06,1.13) |
|  | Dec-04 |  | 1.31 (1.21,1.41) |  | 1.09 (1.05,1.13) |  | 1.15 (1.12,1.19) |
|  | Jan-05 |  | 1.43 (1.33,1.54) |  | 1.12 (1.08,1.16) |  | 1.20 (1.17,1.24) |
|  | Feb-05 |  | 1.56 (1.44,1.68) |  | 1.15 (1.10,1.19) |  | 1.25 (1.21,1.29) |
|  | Mar-05 |  | 1.71 (1.59,1.84) |  | 1.17 (1.12,1.21) |  | 1.30 (1.26,1.34) |
|  | Apr-05 |  | 1.86 (1.73,2.00) |  | 1.19 (1.15,1.23) |  | 1.35 (1.31,1.39) |
|  | May-05 |  | 2.10 (1.96,2.25) |  | 1.19 (1.15,1.23) |  | 1.42 (1.38,1.46) |
|  | Jun-05 |  | 2.18 (2.04,2.34) |  | 1.18 (1.14,1.22) |  | 1.50 (1.46,1.54) |
|  | Jul-05 |  | 2.44 (2.28,2.61) |  | 1.20 (1.16,1.24) |  | 1.56 (1.52,1.61) |
|  | Aug-05 |  | 2.70 (2.52,2.89) |  | 1.21 (1.17,1.25) |  | 1.65 (1.61,1.70) |
|  | Sep-Dec 2005 |  | 2.79 (2.56,3.03) |  | 1.19 (1.14,1.25) |  | 1.67 (1.61,1.73) |

* SEN or an Education Health & Care Plan (S/EHCP) or Action, Action Plus or Support (AAP/S) between reception and Year 6.

**Adjusted for: Sex, parity, size of gestation, mode of delivery, maternal age, ethnic group, quintile of deprivation and expected month of delivery. KS2 results are also adjusted for KS1 attainment.

**Figure S2: Association between gestational age and school attainment* (Black = Key Stage 1, Blue = Key Stage 2), by chronic condition.** Figure shows relative risk, comparing children born at each week of gestation compared with 40 weeks of gestation (within each chronic condition group), adjusted for sex, parity, size for gestation, mode of delivery, maternal age, ethnic group, quintile of deprivation and expected month of delivery. KS2 results are adjusted for KS1 attainment.

*Not achieving Level 2 at Key Stage 1 /Level 4 at Key Stage 2

**Figure S3: Association between gestational age and school attainment*, by size for gestation.** Figure shows relative risk comparing children born at each week of gestation compared with 40 weeks of gestation (within each birth weight category), adjusted for sex, parity, mode of delivery, maternal age, ethnic group, quintile of deprivation and expected month of delivery. KS2 results are adjusted for KS1 attainment.

*Not achieving Level 2 at Key Stage 1 / Level 4 at Key Stage 2

**Table S4: School attainment at Key Stage 1 and attainment / SEN* at Key Stage 2, by gestational age at birth and chronic condition**

|  | **Not achieving expected level (Level 2) at Key Stage 1** | | | **Not achieving expected level (Level 4) at Key Stage 2** | | | **SEN*** | | |
| --- | --- | --- | --- | --- | --- | --- | --- | --- | --- |
| Gestational age group | n (%) | Relative Risk (95% CI) | Adjusted Relative Risk (95% CI) | n (%) | Relative Risk (95% CI) | Adjusted Relative Risk (95% CI) | n (%) | Relative Risk (95% CI) | Adjusted Relative Risk (95% CI) |
| *No chronic conditions* |  |  |  |  |  |  |  |  |  |
| Total | 22329 (7.8) |  |  | 55821 (19.6) |  |  | 86361 (30.3) |  |  |
| 24-32 | 236 (18.5) | 2.52 (2.23,2.83) | 1.90 (1.68,2.13) | 420 (33.6) | 1.73 (1.60,1.87) | 1.21 (1.12,1.30) | 629 (48.0) | 1.67 (1.57,1.76) | 1.42 (1.34,1.51) |
| 32-33 | 234 (12.7) | 1.73 (1.53,1.96) | 1.42 (1.25,1.60) | 519 (28.8) | 1.48 (1.38,1.60) | 1.22 (1.14,1.30) | 787 (42.1) | 1.46 (1.39,1.54) | 1.30 (1.23,1.37) |
| 34-36 | 1294 (11.1) | 1.51 (1.42,1.59) | 1.30 (1.23,1.38) | 2846 (24.8) | 1.28 (1.23,1.32) | 1.11 (1.08,1.15) | 4433 (37.2) | 1.29 (1.26,1.32) | 1.18 (1.15,1.21) |
| 37-38 | 4653 (8.9) | 1.21 (1.17,1.26) | 1.14 (1.10,1.18) | 11 115 (21.7) | 1.12 (1.09,1.14) | 1.06 (1.04,1.09) | 17 336 (32.5) | 1.13 (1.11,1.15) | 1.09 (1.07,1.11) |
| 39 | 4886 (7.9) | 1.07 (1.03,1.11) | 1.04 (1.01,1.08) | 12 304 (20.2) | 1.04 (1.02,1.06) | 1.04 (1.01,1.05) | 19 225 (30.3) | 1.05 (1.04,1.07) | 1.04 (1.02,1.06) |
| 40 | 5989 (7.4) | **Ref** | **Ref** | 15 453 (19.4) | **Ref** | **Ref** | 23 906 (28.8) | **Ref** | **Ref** |
| 41-43 | 5037 (7.3) | 0.99 (0.96,1.03) | 1.03 (0.99,1.07) | 13 164 (19.5) | 1.01 (0.99,1.03) | 1.01 (0.99,1.03) | 20 045 (28.5) | 0.99 (0.97,1.01) | 1.01 (0.99,1.02) |
| *Any chronic condition* |  |  |  |  |  |  |  |  |  |
| Total | 3428 (16.0) |  |  | 6329 (29.6) |  |  | 9584 (44.8) |  |  |
| 24-32 | 252 (31.1) | 2.29 (2.02,2.58) | 1.84 (1.61,2.11) | 397 (50.3) | 1.85 (1.70,2.00) | 1.24 (1.14,1.34) | 546 (65.8) | 1.64 (1.54,1.73) | 1.43 (1.34,1.53) |
| 32-33 | 103 (29.0) | 2.13 (1.79,2.54) | 1.77 (1.48,2.12) | 134 (39.5) | 1.46 (1.27,1.67) | 1.03 (0.92,1.16) | 207 (57.8) | 1.44 (1.31,1.58) | 1.29 (1.18,1.42) |
| 34-36 | 321 (22.7) | 1.67 (1.48,1.88) | 1.50 (1.34,1.69) | 531 (38.1) | 1.40 (1.30,1.52) | 1.12 (1.04,1.20) | 767 (52.6) | 1.31 (1.23,1.39) | 1.23 (1.16,1.30) |
| 37-38 | 811 (18.1) | 1.33 (1.21,1.46) | 1.26 (1.15,1.39) | 1418 (32.2) | 1.19 (1.11,1.26) | 1.06 (1.01,1.12) | 2198 (48.1) | 1.19 (1.14,1.25) | 1.16 (1.11,1.21) |
| 39 | 646 (15.0) | 1.10 (1.00,1.21) | 1.08 (0.98,1.20) | 1245 (29.4) | 1.08 (1.01,1.15) | 1.04 (0.99,1.10) | 1907 (43.2) | 1.07 (1.02,1.12) | 1.07 (1.02,1.12) |
| 40 | 724 (13.6) | **Ref** | **Ref** | 1419 (27.2) | **Ref** | **Ref** | 2180 (40.3) | **Ref** | **Ref** |
| 41-43 | 571 (13.4) | 0.98 (0.89,1.09) | 1.01 (0.91,1.12) | 1185 (28.4) | 1.04 (0.98,1.11) | 1.05 (0.99,1.11) | 1779 (40.8) | 1.01 (0.97,1.06) | 1.02 (0.97,1.07) |
| *Cardiovascular* |  |  |  |  |  |  |  |  |  |
| Total | 634 (29.9) |  |  | 942 (44.4) |  |  | 1257 (59.2) |  |  |
| 24-32 | 101 (35.1) | 1.50 (1.19,1.91) | 1.42 (1.10,1.83) | 154 (55.4) | 1.43 (1.21,1.68) | 1.21 (1.04,1.41) | 200 (68.0) | 1.36 (1.20,1.54) | 1.23 (1.07,1.41) |
| 32-33 | 28 (42.4) | 1.82 (1.30,2.54) | 1.70 (1.21,2.39) | 34 (55.7) | 1.44 (1.11,1.85) | 1.10 (0.87,1.39) | 48 (71.6) | 1.43 (1.19,1.71) | 1.30 (1.08,1.55) |
| 34-36 | 90 (43.1) | 1.85 (1.46,2.34) | 1.67 (1.32,2.11) | 130 (62.2) | 1.60 (1.36,1.89) | 1.19 (1.03,1.38) | 162 (73.3) | 1.46 (1.29,1.66) | 1.33 (1.17,1.51) |
| 37-38 | 152 (32.5) | 1.40 (1.12,1.74) | 1.32 (1.06,1.64) | 216 (47.5) | 1.22 (1.04,1.43) | 1.09 (0.95,1.24) | 292 (61.3) | 1.22 (1.09,1.38) | 1.18 (1.05,1.33) |
| 39 | 83 (25.6) | 1.10 (0.85,1.42) | 1.10 (0.86,1.42) | 135 (42.7) | 1.10 (0.92,1.31) | 1.04 (0.90,1.21) | 185 (56.1) | 1.12 (0.98,1.28) | 1.11 (0.97,1.27) |
| 40 | 94 (23.3) | **Ref** | **Ref** | 153 (38.8) | **Ref** | **Ref** | 204 (50.1) | **Ref** | **Ref** |
| 41-43 | 86 (27.0) | 1.16 (0.90,1.49) | 1.18 (0.92,1.51) | 120 (39.1) | 1.01 (0.84,1.21) | 0.95 (0.82,1.11) | 166 (50.6) | 1.01 (0.87,1.17) | 1.03 (0.90,1.18) |
| *Respiratory* |  |  |  |  |  |  |  |  |  |
| Total | 694 (17.2) |  |  | 1285 (31.8) |  |  | 2002 (49.5) |  |  |
| 24-32 | 143 (37.0) | 2.95 (2.38,3.66) | 2.56 (2.01,3.24) | 213 (57.4) | 2.20 (1.91,2.53) | 1.31 (1.13,1.52) | 288 (72.7) | 1.64 (1.50,1.80) | 1.45 (1.31,1.61) |
| 32-33 | 21 (26.9) | 2.15 (1.44,3.22) | 1.79 (1.20,2.68) | 31 (41.3) | 1.58 (1.18,2.11) | 1.09 (0.85,1.39) | 47 (60.3) | 1.36 (1.12,1.65) | 1.21 (1.01,1.47) |
| 34-36 | 54 (20.5) | 1.64 (1.23,2.20) | 1.60 (1.19,2.14) | 91 (34.7) | 1.33 (1.09,1.62) | 1.06 (0.89,1.27) | 146 (53.9) | 1.22 (1.07,1.39) | 1.19 (1.04,1.35) |
| 37-38 | 155 (18.7) | 1.49 (1.20,1.86) | 1.44 (1.15,1.80) | 268 (32.8) | 1.26 (1.08,1.45) | 1.07 (0.94,1.22) | 431 (50.8) | 1.15 (1.04,1.26) | 1.12 (1.02,1.24) |
| 39 | 101 (13.2) | 1.06 (0.82,1.35) | 1.05 (0.82,1.34) | 224 (29.7) | 1.14 (0.98,1.33) | 1.09 (0.95,1.25) | 352 (44.8) | 1.01 (0.91,1.13) | 1.01 (0.92,1.12) |
| 40 | 117 (12.5) | **Ref** | **Ref** | 241 (26.1) | **Ref** | **Ref** | 423 (44.2) | **Ref** | **Ref** |
| 41-43 | 103 (14.9) | 1.19 (0.93,1.52) | 1.22 (0.96,1.55) | 217 (32.1) | 1.23 (1.05,1.43) | 1.13 (0.99,1.30) | 315 (44.5) | 1.01 (0.90,1.12) | 1.00 (0.90,1.11) |
| *Metabolic* |  |  |  |  |  |  |  |  |  |
| Total | 1449 (13.8) |  |  | 2850 (27.1) |  |  | 4395 (41.8) |  |  |
| 24-32 | 71 (31.4) | 2.77 (2.22,3.45) | 2.23 (1.75,2.83) | 115 (53.2) | 2.14 (1.86,2.47) | 1.32 (1.14,1.52) | 152 (65.0) | 1.73 (1.56,1.93) | 1.50 (1.33,1.69) |
| 32-33 | 42 (26.9) | 2.37 (1.80,3.14) | 2.01 (1.51,2.68) | 53 (36.1) | 1.45 (1.16,1.82) | 1.02 (0.84,1.24) | 78 (50.0) | 1.33 (1.13,1.57) | 1.26 (1.07,1.49) |
| 34-36 | 130 (18.8) | 1.66 (1.37,2.00) | 1.49 (1.24,1.80) | 239 (35.3) | 1.42 (1.26,1.60) | 1.16 (1.04,1.29) | 350 (49.3) | 1.32 (1.20,1.44) | 1.23 (1.12,1.34) |
| 37-38 | 342 (15.4) | 1.36 (1.18,1.57) | 1.30 (1.12,1.50) | 631 (29.0) | 1.17 (1.06,1.28) | 1.07 (0.98,1.16) | 1012 (44.9) | 1.20 (1.12,1.28) | 1.17 (1.10,1.25) |
| 39 | 315 (14.2) | 1.25 (1.08,1.45) | 1.24 (1.07,1.43) | 604 (27.7) | 1.12 (1.01,1.23) | 1.04 (0.96,1.13) | 947 (41.6) | 1.11 (1.04,1.19) | 1.12 (1.04,1.19) |
| 40 | 302 (11.3) | **Ref** | **Ref** | 652 (24.8) | **Ref** | **Ref** | 1013 (37.5) | **Ref** | **Ref** |
| 41-43 | 247 (11.5) | 1.01 (0.87,1.19) | 1.05 (0.90,1.23) | 556 (26.4) | 1.06 (0.96,1.17) | 1.05 (0.97,1.15) | 843 (38.6) | 1.03 (0.96,1.11) | 1.04 (0.97,1.12) |
| *Neurological* |  |  |  |  |  |  |  |  |  |
| Total | 1441 (27.7) |  |  | 2107 (40.5) |  |  | 3037 (58.4) |  |  |
| 24-32 | 132 (39.2) | 1.64 (1.38,1.93) | 1.34 (1.12,1.62) | 186 (56.4) | 1.48 (1.32,1.67) | 1.17 (1.04,1.30) | 258 (75.0) | 1.44 (1.33,1.57) | 1.33 (1.21,1.45) |
| 32-33 | 47 (39.2) | 1.64 (1.28,2.09) | 1.36 (1.06,1.74) | 53 (45.3) | 1.19 (0.96,1.48) | 0.95 (0.81,1.11) | 88 (71.5) | 1.38 (1.22,1.56) | 1.27 (1.12,1.44) |
| 34-36 | 141 (39.4) | 1.65 (1.40,1.94) | 1.47 (1.25,1.73) | 191 (53.5) | 1.41 (1.25,1.59) | 1.09 (0.99,1.20) | 250 (66.1) | 1.27 (1.16,1.39) | 1.22 (1.11,1.33) |
| 37-38 | 372 (34.0) | 1.42 (1.25,1.62) | 1.34 (1.17,1.53) | 508 (47.3) | 1.24 (1.13,1.37) | 1.07 (0.99,1.15) | 724 (64.4) | 1.24 (1.16,1.33) | 1.21 (1.13,1.30) |
| 39 | 249 (25.1) | 1.05 (0.91,1.22) | 1.04 (0.90,1.20) | 389 (39.9) | 1.05 (0.95,1.17) | 1.05 (0.97,1.15) | 560 (54.8) | 1.06 (0.98,1.14) | 1.06 (0.98,1.14) |
| 40 | 284 (23.9) | **Ref** | **Ref** | 442 (38.0) | **Ref** | **Ref** | 634 (52.0) | **Ref** | **Ref** |
| 41-43 | 216 (22.5) | 0.94 (0.80,1.10) | 0.96 (0.83,1.12) | 338 (35.7) | 0.94 (0.84,1.05) | 0.97 (0.89,1.06) | 523 (52.7) | 1.02 (0.94,1.10) | 1.02 (0.94,1.10) |

* Special Educational Needs (SEN) or an Education Health & Care Plan (S/EHCP) or Action, Action Plus or Support (AAP/S)) between reception and Year 6.

**Adjusted for: Sex, parity, size for gestation, mode of delivery, maternal age, ethnic group, quintile of deprivation and expected month of delivery. KS2 additionally adjusted for KS1 results.

**Table S5: Population attributable fractions for not achieving the expected levels at Key Stage 1 and Key Stage 2, and SEN*.**

|  | **Proportion exposed** | **Attributable proportion among exposed** | **Population attributable fraction (PAF)** |
| --- | --- | --- | --- |
| *Not achieving expected level at Key Stage 1* | | | |
| Preterm birth (24-37 weeks) | 0.06 | 0.41 | 0.039 |
| Early term birth (37-38 weeks) | 0.19 | 0.14 | 0.029 |
| Any chronic condition by age 2 | 0.07 | 0.51 | 0.068 |
|  |  |  |  |
| *Not achieving expected level at Key Stage 2* |  |  |  |
| Preterm birth (24-37 weeks) | 0.06 | 0.27 | 0.021 |
| Early term birth (37-38 weeks) | 0.19 | 0.08 | 0.016 |
| Any chronic condition by age 2 | 0.07 | 0.34 | 0.034 |
|  |  |  |  |
| *SEN** |  |  |  |
| Preterm birth (24-37 weeks) | 0.06 | 0.26 | 0.020 |
| Early term birth (37-38 weeks) | 0.19 | 0.09 | 0.018 |
| Any chronic condition by age 2 | 0.07 | 0.32 | 0.032 |

* Special Educational Needs (SEN) or an Education Health & Care Plan (S/EHCP) or Action, Action Plus or Support (AAP/S) between reception and Year 6.

Notes: The Population Attributable Fraction (PAF) is calculated by dividing the unadjusted Population Attributable Risk (PAR) by the prevalence of risk in the population. It describes the contribution of a risk factor (chronic condition, preterm birth, early term birth) to the burden of an outcome (academic attainment or SEN) under the assumption of a causal association. For example, we estimate that 3.2% of SEN is attributable to chronic condition by age 2. PAR is defined as the excess rate of disease in the total population (exposed and unexposed) that is attributable to the exposure. It is calculated by subtracting the risk in the unexposed population from the risk in the population (exposed and unexposed). The Attributable proportion among exposed represents the expected reduction in an outcome if the exposure could be removed, assuming a causal association. It is calculated as the fraction of a) the risk for the exposed population minus risk for unexposed population, and b) the risk for the exposed population.

**Table S6:** **School attainment at Key Stage 1 and attainment / SEN* at Key Stage 2, by gestational age at birth, using multiple imputation (10 imputations)**

|  | Not achieving expected level (Level 2) at Key Stage 1 | | Not achieving expected level (Level 4) at Key Stage 2 | | SEN* | |
| --- | --- | --- | --- | --- | --- | --- |
| Week of gestation | Crude Relative Risk (95% CI) | Adjusted** Relative Risk (95% CI) | Crude Relative Risk (95% CI) | Adjusted** Relative Risk (95% CI) | Crude Relative Risk (95% CI) | Adjusted** Relative Risk (95% CI) |
| 24 | 6.04 (4.57,7.97) | 4.65 (3.54,6.10) | 2.74 (2.13,3.53) | 1.21 (1.00,1.47) | 2.65 (2.31,3.04) | 2.22 (1.88,2.62) |
| 25 | 3.84 (2.78,5.29) | 2.66 (1.92,3.69) | 2.76 (2.27,3.36) | 1.48 (1.23,1.77) | 2.47 (2.17,2.81) | 1.93 (1.69,2.20) |
| 26 | 3.76 (2.94,4.81) | 2.66 (2.09,3.39) | 2.49 (2.11,2.93) | 1.38 (1.18,1.61) | 2.26 (2.02,2.53) | 1.88 (1.67,2.11) |
| 27 | 3.64 (2.94,4.52) | 2.38 (1.93,2.92) | 2.07 (1.75,2.44) | 1.14 (0.99,1.30) | 1.97 (1.76,2.22) | 1.51 (1.34,1.69) |
| 28 | 3.44 (2.87,4.11) | 2.35 (1.98,2.80) | 2.21 (1.96,2.51) | 1.23 (1.10,1.38) | 1.90 (1.72,2.09) | 1.50 (1.36,1.65) |
| 29 | 2.59 (2.12,3.16) | 1.90 (1.56,2.31) | 1.87 (1.64,2.14) | 1.24 (1.10,1.39) | 1.71 (1.55,1.89) | 1.40 (1.27,1.54) |
| 30 | 2.31 (1.92,2.78) | 1.70 (1.41,2.04) | 1.82 (1.62,2.04) | 1.26 (1.14,1.40) | 1.68 (1.54,1.83) | 1.38 (1.27,1.50) |
| 31 | 2.32 (1.96,2.74) | 1.80 (1.51,2.13) | 1.67 (1.49,1.87) | 1.20 (1.08,1.33) | 1.67 (1.54,1.81) | 1.42 (1.31,1.54) |
| 32 | 1.89 (1.62,2.21) | 1.50 (1.29,1.75) | 1.47 (1.33,1.62) | 1.14 (1.05,1.25) | 1.53 (1.42,1.64) | 1.32 (1.23,1.42) |
| 33 | 1.90 (1.67,2.15) | 1.57 (1.39,1.77) | 1.50 (1.39,1.62) | 1.19 (1.10,1.27) | 1.48 (1.38,1.57) | 1.31 (1.23,1.39) |
| 34 | 1.56 (1.40,1.72) | 1.30 (1.18,1.44) | 1.32 (1.23,1.42) | 1.12 (1.06,1.19) | 1.36 (1.29,1.43) | 1.21 (1.15,1.28) |
| 35 | 1.41 (1.29,1.54) | 1.23 (1.13,1.35) | 1.26 (1.20,1.33) | 1.11 (1.07,1.16) | 1.34 (1.28,1.39) | 1.22 (1.18,1.27) |
| 36 | 1.49 (1.40,1.58) | 1.33 (1.25,1.41) | 1.25 (1.20,1.30) | 1.09 (1.06,1.13) | 1.27 (1.24,1.31) | 1.18 (1.15,1.21) |
| 37 | 1.31 (1.26,1.37) | 1.22 (1.16,1.27) | 1.16 (1.13,1.20) | 1.07 (1.04,1.10) | 1.19 (1.16,1.21) | 1.13 (1.11,1.16) |
| 38 | 1.18 (1.14,1.22) | 1.13 (1.09,1.17) | 1.10 (1.08,1.12) | 1.05 (1.03,1.07) | 1.13 (1.11,1.14) | 1.09 (1.08,1.11) |
| 39 | 1.07 (1.04,1.11) | 1.05 (1.02,1.09) | 1.05 (1.03,1.07) | 1.03 (1.01,1.05) | 1.06 (1.04,1.07) | 1.05 (1.03,1.06) |
| **40** | **Ref** | **Ref** | **Ref** | **Ref** | **Ref** | **Ref** |
| 41 | 0.98 (0.94,1.01) | 1.00 (0.97,1.04) | 1.00 (0.98,1.02) | 1.01 (0.99,1.02) | 0.97 (0.96,0.99) | 0.99 (0.97,1.00) |
| 42 | 0.99 (0.94,1.05) | 1.06 (1.00,1.12) | 0.99 (0.96,1.03) | 1.01 (0.99,1.04) | 0.97 (0.95,0.99) | 1.01 (0.98,1.03) |
| 43 | 0.95 (0.85,1.07) | 1.02 (0.91,1.13) | 0.99 (0.91,1.07) | 1.01 (0.94,1.08) | 0.92 (0.86,0.98) | 0.96 (0.90,1.02) |

* Special Educational Needs (SEN) or an Education Health & Care Plan (S/EHCP) or Action, Action Plus or Support (AAP/S)) between reception and Year 6.

**Adjusted for: Sex, parity, size of gestation, mode of delivery, maternal age, ethnic group, quintile of deprivation and expected month of delivery. KS2 additionally adjusted for KS1 results.

Notes: We used multiple imputation by chained equations. We used a truncated regression for continuous gestational age values, truncated at 24 and 43 months. Multinomial logit models were used for categorical maternal age, parity/previous births, ethnic group, mode of delivery. Order logit models were used for size for gestation, quintile of index of multiple deprivation, and month of expected delivery date. Logit models were used for SEN and sex. Based on these models, we created 10 datasets where missing data were imputed. We averaged results across these imputed datasets using Rubin’s rules.

**Table S7:** **School attainment at Key Stage 1 and attainment / SEN* at Key Stage 2, by gestational age at birth, for spontaneous vaginal births only**

|  | Not achieving expected level (Level 2) at Key Stage 1 | | Not achieving expected level (Level 4) at Key Stage 2 | | SEN* | |
| --- | --- | --- | --- | --- | --- | --- |
| Week of gestation | Crude Relative Risk (95% CI) | Adjusted** Relative Risk (95% CI) | Crude Relative Risk (95% CI) | Adjusted** Relative Risk (95% CI) | Crude Relative Risk (95% CI) | Adjusted** Relative Risk (95% CI) |
| 24 | 5.89 (4.21,8.24) | 4.74 (3.34,6.74) | 2.92 (2.23,3.83) | 2.68 (2.03,3.53) | 2.63 (2.25,3.08) | 2.29 (1.87,2.79) |
| 25 | 4.37 (3.04,6.29) | 3.12 (2.10,4.62) | 2.62 (2.04,3.37) | 2.27 (1.76,2.93) | 2.50 (2.15,2.91) | 2.05 (1.76,2.38) |
| 26 | 4.26 (3.08,5.90) | 3.14 (2.29,4.31) | 2.43 (1.93,3.07) | 2.22 (1.74,2.82) | 2.20 (1.87,2.58) | 1.89 (1.62,2.22) |
| 27 | 3.39 (2.28,5.03) | 2.30 (1.61,3.28) | 1.52 (1.06,2.19) | 1.27 (0.90,1.80) | 1.60 (1.24,2.05) | 1.23 (0.96,1.58) |
| 28 | 3.21 (2.36,4.38) | 2.15 (1.58,2.91) | 2.03 (1.64,2.52) | 1.71 (1.37,2.13) | 1.93 (1.66,2.24) | 1.50 (1.28,1.76) |
| 29 | 2.90 (2.13,3.95) | 2.27 (1.69,3.05) | 1.89 (1.52,2.34) | 1.68 (1.36,2.07) | 1.73 (1.47,2.03) | 1.40 (1.19,1.65) |
| 30 | 1.80 (1.24,2.60) | 1.36 (0.95,1.95) | 1.88 (1.55,2.28) | 1.68 (1.39,2.03) | 1.68 (1.44,1.94) | 1.35 (1.17,1.57) |
| 31 | 1.97 (1.45,2.69) | 1.63 (1.19,2.22) | 1.50 (1.23,1.84) | 1.39 (1.12,1.72) | 1.50 (1.30,1.74) | 1.32 (1.14,1.53) |
| 32 | 1.81 (1.42,2.32) | 1.53 (1.20,1.95) | 1.55 (1.33,1.80) | 1.45 (1.25,1.68) | 1.52 (1.36,1.70) | 1.33 (1.20,1.48) |
| 33 | 1.91 (1.58,2.30) | 1.59 (1.32,1.90) | 1.57 (1.40,1.76) | 1.45 (1.30,1.63) | 1.49 (1.36,1.62) | 1.31 (1.21,1.43) |
| 34 | 1.52 (1.30,1.77) | 1.32 (1.13,1.54) | 1.25 (1.13,1.38) | 1.18 (1.07,1.30) | 1.30 (1.21,1.40) | 1.17 (1.09,1.25) |
| 35 | 1.32 (1.16,1.50) | 1.19 (1.04,1.35) | 1.25 (1.15,1.34) | 1.20 (1.11,1.29) | 1.30 (1.23,1.37) | 1.19 (1.13,1.26) |
| 36 | 1.61 (1.49,1.75) | 1.41 (1.31,1.53) | 1.29 (1.22,1.36) | 1.23 (1.17,1.29) | 1.25 (1.21,1.30) | 1.15 (1.10,1.19) |
| 37 | 1.31 (1.23,1.39) | 1.19 (1.11,1.26) | 1.17 (1.12,1.21) | 1.13 (1.09,1.17) | 1.17 (1.14,1.21) | 1.10 (1.07,1.13) |
| 38 | 1.22 (1.17,1.28) | 1.15 (1.10,1.20) | 1.11 (1.08,1.14) | 1.10 (1.07,1.13) | 1.14 (1.11,1.16) | 1.09 (1.07,1.12) |
| 39 | 1.08 (1.04,1.12) | 1.05 (1.01,1.09) | 1.04 (1.01,1.06) | 1.04 (1.01,1.06) | 1.06 (1.04,1.08) | 1.05 (1.03,1.06) |
| 40 | **Ref** | **Ref** | **Ref** | **Ref** | **Ref** | **ref** |
| 41 | 0.99 (0.95,1.04) | 1.02 (0.97,1.06) | 1.02 (0.99,1.04) | 1.01 (0.99,1.04) | 0.99 (0.97,1.01) | 1.00 (0.98,1.01) |
| 42 | 1.08 (1.00,1.18) | 1.15 (1.06,1.24) | 1.04 (0.99,1.09) | 1.06 (1.01,1.11) | 1.05 (1.01,1.08) | 1.07 (1.03,1.10) |
| 43 | 1.13 (0.90,1.42) | 1.08 (0.86,1.36) | 1.18 (1.03,1.34) | 1.13 (0.99,1.28) | 1.12 (1.01,1.24) | 1.08 (0.98,1.19) |

* Special Educational Needs (SEN) or an Education Health & Care Plan (S/EHCP) or Action, Action Plus or Support (AAP/S)) between reception and Year 6.

**Adjusted for: Sex, parity, size of gestation, mode of delivery, maternal age, ethnic group, quintile of deprivation and expected month of delivery. KS2 additionally adjusted for KS1 results.

**Figure S4:** **School attainment at Key Stage 2*, by gestational age at birth, with and without adjusting for Key Stage 1 attainment.** Figure shows relative risk comparing children born at each week of gestation compared with 40 weeks of gestation, adjusted for sex, parity, mode of delivery, maternal age, ethnic group, quintile of deprivation and expected month of delivery.

*Not achieving Level 4 at Key Stage 2
